# An anionic ligand snap-locks a long-range interaction in a magnesium-folded riboswitch

**DOI:** 10.1038/s41467-021-27827-y

**Published:** 2022-01-11

**Authors:** Rajeev Yadav, Julia R. Widom, Adrien Chauvier, Nils G. Walter

**Affiliations:** 1 Single Molecule Analysis Group, Department of Chemistry and Center for RNA Biomedicine, University of Michigan, Ann Arbor, MI 48109 USA; 2 Present Address: Department of Physics and Astronomy, Michigan State University, East Lansing, MI 48824 USA; 3 Present Address: Department of Chemistry and Biochemistry, University of Oregon, Eugene, OR 97403 USA

**Keywords:** Single-molecule biophysics, Small RNAs

## Abstract

The archetypical transcriptional *crcB* fluoride riboswitch from *Bacillus cereus* is an intricately structured non-coding RNA element enhancing gene expression in response to toxic levels of fluoride. Here, we used single molecule FRET to uncover three dynamically interconverting conformations appearing along the transcription process: two distinct undocked states and one pseudoknotted docked state. We find that the fluoride anion specifically snap-locks the magnesium-induced, dynamically docked state. The long-range, nesting, single base pair A40-U48 acts as the main linchpin, rather than the multiple base pairs comprising the pseudoknot. We observe that the proximally paused RNA polymerase further fine-tunes the free energy to promote riboswitch docking. Finally, we show that fluoride binding at short transcript lengths is an early step toward partitioning folding into the docked conformation. These results reveal how the anionic fluoride ion cooperates with the magnesium-associated RNA to govern regulation of downstream genes needed for fluoride detoxification of the cell.

## Abstract (web summary)

A single molecule fluorescence study revealed three dynamically interconverting conformations of the fluoride riboswitch from *Bacillus cereus*, where an anionic ligand snap-locks a docked conformation through a long-range interaction necessary for downstream gene regulation.

## Introduction

Riboswitches are structured noncoding RNA elements typically found in the 5′ untranslated regions of bacterial messenger RNAs^1–5^. They regulate gene expression, most often at the level of transcription and translation, through the binding of specific ligands that induce changes in riboswitch structure. A typical riboswitch comprises a highly structured aptamer (receptor) domain and a downstream regulatory domain (expression platform); binding of the ligand to the aptamer domain induces a conformational switch in the expression platform that directs downstream gene expression. So far over 40 classes of riboswitches have been identified, sensing diverse ligands including metabolites, enzyme cofactors, and metal ions^3,6–9^. The critical role of riboswitches in bacterial gene regulation makes them an attractive target for the development of new antibiotic therapies^10^.

**Fig. 1:**
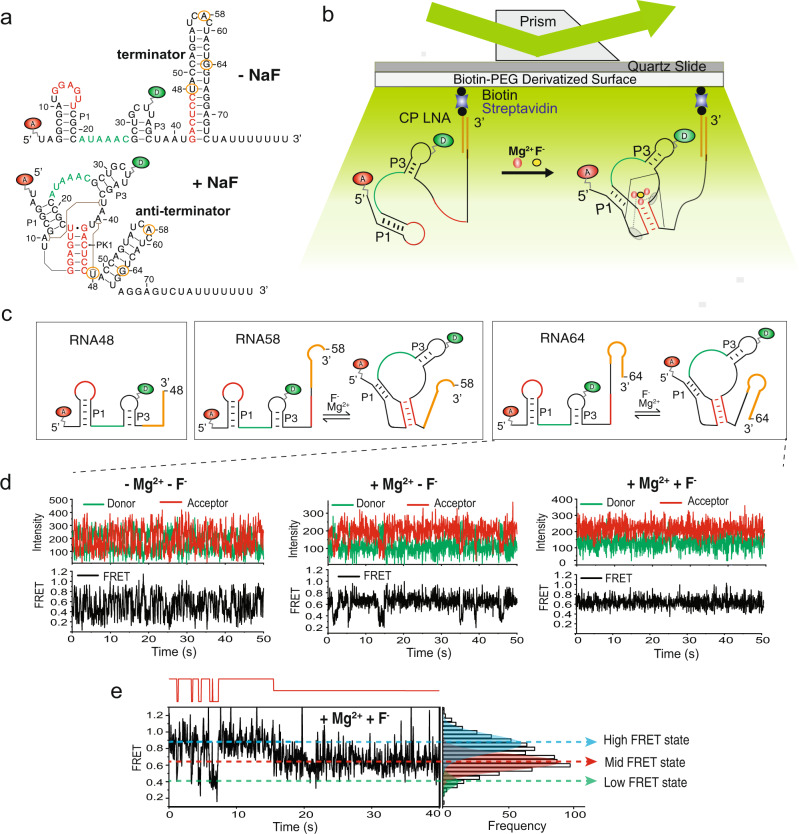
smFRET analysis of the *Bacillus cereus crcB* fluoride riboswitch. **a** Structure of the fluoride riboswitch in the absence and presence of fluoride. The circled nucleotides show the 3′ ends of the constructs used for smFRET. The positions of donor and acceptor are shown by green and red ovals labeled D and A, respectively. **b** Experimental smFRET setup. The RNA is immobilized through a biotinylated LNA capture probe. **c** The different lengths of RNA used for smFRET. The orange sequence at the 3′ end of the riboswitch is complementary to the CP LNA (or bubble DNA, in experiments performed with RNAP as shown in Fig. 4). The red and green segments are a guide to the eye to compare the riboswitch structure in the absence and presence of F^-^, analogous to the color scheme used in panel a. **d** Representative smFRET traces for RNA64 in the absence of Mg^2+^ and F^-^ (left), in the presence of only Mg^2+^ (middle), and in the presence of both Mg^2+^ and F^-^ (right). **e** Example of an RNA64 smFRET trace that transitions between all three FRET states (observed in 5–10% of traces in the presence of Mg^2+^ only and Mg^2+^ plus F^-^). The HMM fit is shown in red above the trace and the FRET distribution of the trace, fitted with three Gaussian peaks, is shown on the right.

Fluoride riboswitches, also known as *crcB* motif RNAs, are a family of transcriptional riboswitches present in many bacteria and archaea^11^. On recognition of detrimental levels of fluoride (F^-^), they activate the expression of genes that encode fluoride-sensitive transporters and enzymes such as enolase and formate-hydrogenlyase to detoxify the cell^11,12^. We focus on the archetypical mesophilic *Bacillus cereus* (*B. cereus*) fluoride riboswitch, which contains an intrinsic terminator that becomes disrupted upon F^-^ binding (Fig. 1a). The crystal structure of the ligand-bound thermophilic aptamer of the ﻿*Thermotoga petrophila* fluoride riboswitch shows a pseudoknot-structured docked conformation stabilized by long-range interactions, with the F^-^ binding pocket surrounded by three Mg^2+^ ions coordinated with backbone phosphate groups and water molecules^13^. NMR spectroscopy of the *B. cereus* fluoride riboswitch showed that it adopts a similar tertiary structure in solution both in ligand-bound and ligand-free form, where the ligand allosterically induces the formation of a reverse Hoogsteen A40-U48 base pair at the interface of the aptamer and expression platform^14^. These structural studies were performed at equilibrium, but RNA must fold during transcription where it might function before it is fully transcribed, with the RNA polymerase (RNAP) potentially pausing to assist^6,15,16^.

Various methods have been used to understand co-transcriptional folding of RNA including single-molecule fluorescence resonance energy transfer (smFRET);^17–19^ optical-trapping;^6^ and selective 2′-hydroxyl acylation analyzed by primer extension sequencing (SHAPE-seq)^20–22^. Applying SHAPE-seq to the *B. cereus* fluoride riboswitch with RNAP stalled at each nucleotide position, in particular, revealed ligand-dependent delays in terminator formation and identified the point at which the outcome of transcription is determined^20^. Despite these advances, missing is a dynamic conformational analysis of global RNA structure at functionally relevant transcription points, as signified by either RNA folding topology or intrinsic RNAP pausing, as potential key forces in the mechanism of ligand-guided gene regulation^23^.

Here, we used smFRET to determine the conformational dynamics of the *B. cereus* fluoride riboswitch in dependence of both Mg^2+^ and F^-^, first in isolation, then in the presence of bacterial RNAP. We find that the riboswitch in the presence of Mg^2+^ folds into a pseudoknotted docked conformation, previously observed by NMR spectroscopy^14^, that still transiently visits two other, less intricately folded states; upon addition of F^-^, the docked structure becomes stably snap-locked by engaging the long-range linchpin A40-U48 base pair. The proximity of RNAP in a downstream elongation complex facilitates the folding of the aptamer into this stably docked conformation. Overall, our results reveal the importance of dynamic RNA docking as a path toward conformational capture by an anionic ligand, aided by RNAP.

## Results

### smFRET analysis reveals three conformational states of the fluoride riboswitch

In the present study, we used smFRET to investigate the fluoride riboswitch in isolation (Fig. 1b) and as a component of transcription elongation complexes (ECs), comparing the equilibrium populations of accessible conformational states and their kinetic parameters. Given that riboswitch properties and interactions within the transcription machinery will evolve as additional RNA sequence is synthesized in 5’-to-3’ direction^16^, we prepared a series of three riboswitch variants—termed RNA48, RNA58 and RNA64 in reference to their lengths—by splint ligation. The ligated 5′ RNA segment contains both donor and acceptor fluorophores, placed to interrogate aptamer pseudoknot formation by smFRET, whereas the 3′ segment entails nucleotides that can be surface captured and mimic the 3′ most 7-10 nucleotides of the transcript in the RNA/DNA transcription hybrid not available for folding (Fig. 1c). The variant of shortest length (RNA48) represents the RNA produced at a ligand-dependent pause position that we observed using in vitro transcription assays (Supplementary Fig. 1). By comparison, in RNA58 the aptamer is fully synthesized and can fold into the pseudoknotted conformation, however, with its 3’ segment still residing in the exit channel of RNAP^20^. Finally, in RNA64 the aptamer domain has completely emerged from the exit channel and is free to fold. We first characterized this latter RNA as a reference baseline.

To address the effects of both Mg^2+^ and F^-^ ions on the conformation of the complete aptamer domain, we investigated the structural dynamics of RNA64 upon immobilization on polyethylene glycol (PEG)-passivated, streptavidin-coated quartz slides via a biotinylated locked nucleic acid (LNA) capture probe (CP, covering the short anchor sequence downstream shown in orange in Fig. 1b). We used smFRET to monitor changes in the positions of donor and acceptor fluorophores, with the donor placed at position 33 and the acceptor coupled to the 5’ end of the riboswitch. Based on hidden Markov modeling (HMM), we found the traces to exhibit three FRET states, of low- (~0.4), mid- (~0.6–0.7) and high-FRET value (~0.8–0.85; Fig. 1d, e, and Supplementary Fig. 2). Upon addition of Mg^2+^ and F^-^, most molecules become stabilized in the mid-FRET state (Fig. 1d, right; Fig. 1e shows a trace where this occurs during observation), with an estimated inter-fluorophore distance of 46-50 Å that is very close to the distance observed in the crystal structure (50 Å; based on PDB ID 4ENC)^13^. These observations are consistent with the notion that the mid-FRET state represents the previously characterized, compact “docked” conformation of the fluoride riboswitch, henceforth referred to as “D” state.

To further characterize the three states we distinguished, we performed a control experiment wherein we blocked the formation of the main pseudoknot (PK1; we note that several different nomenclatures are in use - we here follow reference^20^) using an LNA oligonucleotide complementary to nucleotides A39-U48 of the riboswitch (Supplementary Fig. 3). Independent of Mg^2+^ and F^-^, we observed only the high-FRET state, supporting the notion that, despite representing a structure with proximal fluorophores, this state lacks the pseudoknot and thus docking; we therefore refer to it as “prefolded” (P). By comparison, the low-FRET state of RNA64 is much less compact (as evidenced by the distal fluorophores) and will be denoted as unfolded (U). In the following, we further test these assignments.

### Increasing magnesium induces folding into two distinct D states, first dynamic, then static

**Fig. 2:**
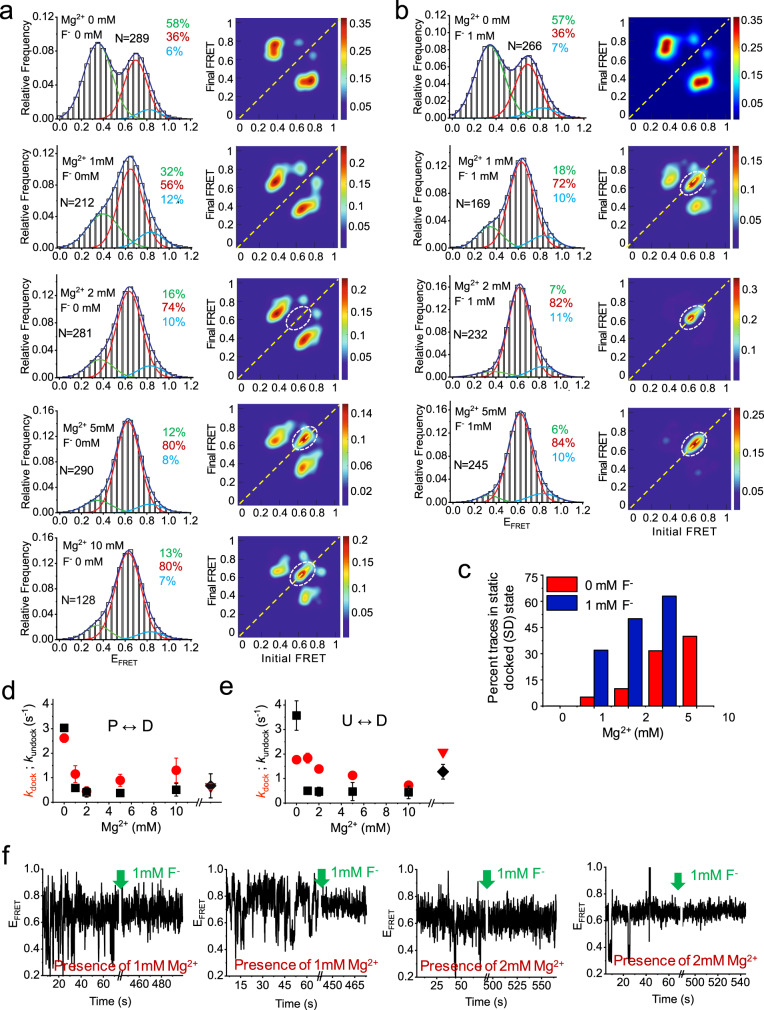
Effect of Mg^2+^ and F^-^ on the folding of the riboswitch (RNA64). FRET histograms and corresponding TODPs in the absence (**a**) and presence (**b**) of F^-^. Histograms are well fitted with three Gaussian peaks, shown in green, red and cyan for the low-, mid- and high-FRET states, respectively, with the cumulative fit shown in blue. The percent population of all fitted peaks are shown in respective colors in each histogram panel, and the number of molecules that were analyzed is indicated by “N”. TODPs represent dynamic traces as ‘off-diagonal’ and static traces as ‘on-diagonal’ features, where the color scale shows the prevalence of each population. **c** Percent of all traces that remain statically in the mid-FRET (docked) state as a function of Mg^2+^concentration in the absence (red) and presence (blue) of 1 mM F^-^. **d**, **e** Kinetics of low- to mid-FRET and high- to mid-FRET transitions as a function of Mg^2+^ concentration. P, U, D stand for prefolded (high-FRET), unfolded (low-FRET) and docked (mid-FRET) states, respectively. The black diamond and red triangle symbols represent rates in 2 mM Mg^2+^ *and* 1 mM F^-^. The error bars are presented as ± standard deviation for *n* = 2 independent experimental datasets. **f** Representative smFRET traces during real-time ligand jump experiments at 1 mM Mg^2+^ (left two traces) and at 2 mM Mg^2+^ (right two traces) before and after the addition of 1 mM F^-^. Upon addition of F^-^ molecules transition from the DD to the SD conformation. The time of F^-^ addition is indicated by the green arrow.

To determine the populations in the three conformational states under different conditions, we generated FRET histograms from 100-300 molecules each and fitted them using a sum of multiple Gaussian peaks (Fig. 2a, b). As expected from our qualitative inspection, the FRET histograms were best fitted with three peaks centered at E_FRET_ ≈ 0.35, 0.69, and 0.83 in the absence of Mg^2+^, and similarly E_FRET_ ≈ 0.35﻿, 0.63, and 0.85 in the presence of 5 mM Mg^2+^. With Mg^2+^, however, the mid-FRET D state becomes more populated, increasing from ~36% in the absence of Mg^2+^ to 56% in 1 mM Mg^2+^, and eventually saturating at 80% at 5 mM Mg^2+^ (Fig. 2a). This Mg^2+^-dependence is fitted well with a Hill equation to yield a half-saturation point of *K*_*1/2*_ ~ 1 mM and a cooperativity coefficient of *n* = 2.1 (Supplementary Fig. 4). Taken together, these observations provide further evidence that the mid-FRET is the Mg^2+^-dependent docked D state and requires multiple Mg^2+^ ions to form.

Next, we generated transition occupancy density plots (TODPs), which show as a heat map the fraction of single-molecule traces that exhibit at least once any given initial-to-final FRET transition^24^. In the presence of nonphysiologically high (10 mM) Mg^2+^, a sub-population of static traces in the mid-FRET (~0.63) state becomes dominant, evidenced on the diagonal in the TODP (Fig. 2a, b). At a more physiological 1 mM Mg^2+^, only 4% of traces reside in this static D state, rising to 30% and 38% in the presence of 5 mM and 10 mM Mg^2+^, respectively (Fig. 2c). These data indicate that the fluoride riboswitch transiently interconverts between docked and undocked conformations in the absence of Mg^2+^, and increasingly adopts a static form of the D state as the Mg^2+^ concentration is increased. The observed dwell time in this static mid-FRET state is limited by photobleaching, which ranges from 5 s to 80 s with a mean time of ~20 s (Supplementary Fig. 5), representing a lower limit of the static D state lifetime. We henceforth call this the stably docked (SD) conformation of the fluoride riboswitch.

Along with the SD population, a substantial portion of molecules still dynamically transition among FRET states as evidenced by the off-diagonal contours in the TODPs (Fig. 2a). We measured rate constants for docking and undocking from both the prefolded (P, high-FRET) and unfolded (U, low-FRET) to the docked (D, mid-FRET) state (Fig. 2d, e, and Supplementary Table 1). For U to D transitions, the docking and undocking rate constants are *k*_dock_ ≈ 1.8 s^−1^ and *k*_undock_ ≈ 3.6 s^−1^ in the absence of Mg^2+^, slowing to 1.4 s^−1^ and 0.5 s^−1^, respectively, in the presence of 2 mM Mg^2+^. The more significant reduction in the *k*_undock_ rate constant upon Mg^2+^ addition shows that the metal ion also promotes this dynamic docked (or DD) conformation. At Mg^2+^ concentrations higher than 1 mM these rate constants remain unchanged (Fig. 2e), suggesting that—in contrast to the SD state population—they plateau at physiological Mg^2+^ concentrations of ~1 mM. That is, physiological Mg^2+^ concentrations induce the riboswitch into the dynamic docking (DD conformation), whereas higher Mg^2+^ concentrations access a distinct, locked SD conformation.

### Fluoride snap-locks the Mg^2+^-bound riboswitch from the DD into the SD state by conformational selection

We next asked how the conformation of the riboswitch changes in response to ligand binding. We did not observe any change in the FRET histograms or TODPs when 1 mM F^-^ was added in the absence of Mg^2+^, indicating that the anionic ligand does not appreciably bind the negatively charged riboswitch (Fig. 2b). In contrast, in the presence of 1 mM and 2 mM Mg^2+^ the addition of 1 mM F^-^ leads to a drastic (~sixfold and ~fivefold over zero F^-^, respectively) increase in the population of the SD conformation (Fig. 2b, c). Our findings indicate that F^-^ locks the Mg^2+^-induced DD conformation into the SD conformation more potently than an increase in Mg^2+^ concentration.

This becomes particularly evident in a ligand-jump experiment^25^ wherein we observe the same set of molecules before and after the addition of F^-^. We find that most molecules respond to the F^-^ jump in both 1 mM and 2 mM Mg^2+^, with DD traces converting directly into the SD state (Fig. 2f). These observations support the notion that the DD state transiently samples a correctly folded docked conformation, wherein the three crystallographically observed^13^ Mg^2+^ ions create a transient, positively charged pocket in the highly negatively-charged RNA. F^-^ then binds to this pocket and “snap-locks” the docked conformation into place, resulting in the observed high population of SD molecules. In light of the two alternate mechanisms of molecular recognition known as conformational selection (CS, “folding first”) or induced-fit (IF, “binding first”)^26,27^, our data suggest that F^-^ only binds to the Mg^2+^-stabilized docked state of the riboswitch and thus follows the CS mechanism.

### The linchpin base-pair A40-U48 is required for the SD state

**Fig. 3:**
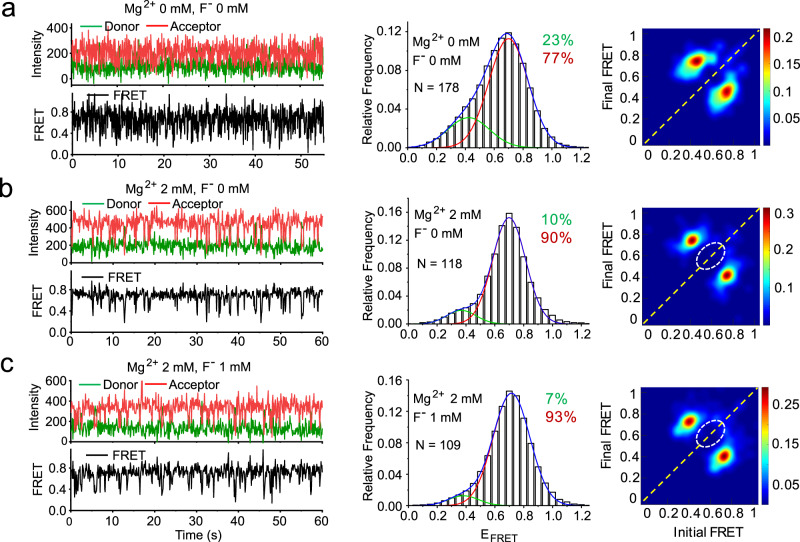
U48A mutation in RNA64 leads to loss of the SD state. Representative smFRET traces, histograms and TODPs for the U48A mutant (**a**) in the absence of Mg^2+^ and F^-^; (**b**) in the presence of 2 mM Mg^2+^ alone; and (**c**) in the presence of both 2 mM Mg^2+^ and 1 mM F^-^. The percent population of all fitted peaks are shown in respective colors in each histogram panel. The dashed white circle in the TODP highlights the absence of the SD state. The number of molecules that were analyzed is indicated by “N”.

The highly conserved reverse Hoogsteen A40-U48 base pair (Fig. 1a) was previously observed by NMR and X-ray crystallography to cap the PK1 helix in the presence of F^-^ and therefore proposed to steer transcription toward anti-termination^13,14^ (note that the latter reference labels it as A37-U45). We therefore asked whether this interaction is important for forming the SD state by disrupting it with a site-specific base mutation, U48A. In the absence of both Mg^2+^ and F^-^, the U48A mutant riboswitch traces are highly dynamic. The population of the low-FRET U state dramatically decreased by ∼threefold in the U48A mutant compared to the U48 wild-type, and we did not observe a distinct P state, perhaps because the DD state was shifted slightly higher in E_FRET_ value, resulting in overlap (Fig. 3a). In the presence of 2 mM Mg^2+^, the U48A mutant resembles the wild-type, with a minor (10%) U state at E_FRET_ ≈ 0.35 and a major (90%) DD state at E_FRET_ ≈ 0.7 (Fig. 3b). Most strikingly, upon addition of F^-^ there was no evidence of on-diagonal SD traces in the TODP (Fig. 3), suggesting that the linchpin base-pair A40-U48 is essential for fluoride-mediated stabilization of the SD state.

The observed low population of the U state in the absence of Mg^2+^ and F^-^ is consistent with the results of our measurements with bases A39 through U48 blocked using a complementary LNA (Supplementary Fig. 3). Like the mutation, this blockage precludes the formation of the A40-U48 base-pair, but it additionally precludes pseudoknot formation and yields the P state almost exclusively. Nearly identical behavior is observed for RNA48, in which the 3′ arm of the pseudoknot is sequestered, again precluding docking and formation of the A40-U48 base-pair. Thus, any disruption to the A40-U48 base-pair significantly disfavors the U state, whether or not the disruption also impacts the pseudoknot. If the pseudoknot is additionally disrupted, only the P state is significantly populated (RNA48 and RNA64 with blocking LNA), whereas the mutation alone allows both the D and P states to remain populated. The SD state is accessible only when both the pseudoknot and the A40-U48 base-pair can form, as in wild-type RNA64 (Fig. 2).

### RNA polymerase stabilizes riboswitch docking

**Fig. 4:**
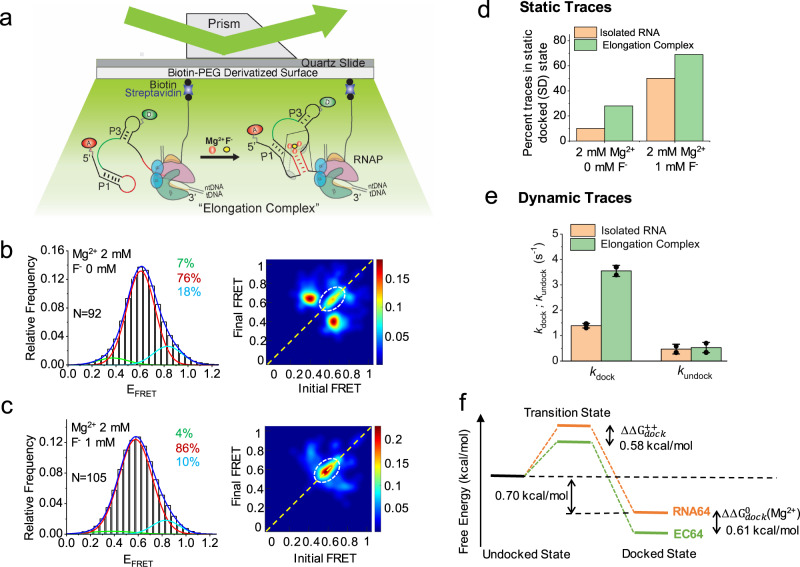
The presence of RNA polymerase favors the docked conformation of RNA64. (**a**) smFRET experimental setup for probing elongation complexes. RNA is immobilized through biotinylated *E. coli* RNAP. (**b, c**) FRET histograms and corresponding TODPs in the presence of only 2 mM Mg^2+^ (**b**) and in the presence of both 2 mM Mg^2+^ and 1 mM F^-^ (**c**). Histograms are well fitted with three Gaussian peaks, shown in green, red and cyan for the low-, mid-, and high-FRET states, respectively, with the cumulative fit shown in blue. The percent population of all fitted peaks are shown in respective colors in each FRET histogram panel, and the number of molecules that were analyzed is indicated by “N”. A dashed white circle highlights the presence of the SD conformation in both TODPs. (**d**) Percent of all traces that remain statically in the mid-FRET (docked) state for isolated RNA (orange) and ECs (green) both in the presence of 2 mM Mg^2+^ only (left) and in the presence of both 2 mM Mg^2+^ and 1 mM F^-^ (right). (**e**) Kinetic parameters for the unfolded-to-folded docked (low- to mid-FRET) transition in the presence of 2 mM Mg^2+^. The error bars are presented as ± standard deviation for n = 2 independent datasets (with the values as *k*_dock_: 1.4 ± 0.1 s^-1^ and *k*_undock_: 0.5 ± 0.2 s^-1^ for isolated RNA and *k*_dock_: 3.6 ± 0.2 s^-1^ and *k*_undock_: 0.5 ± 0.2 s^-1^ for EC). Reliable kinetic parameters could not be obtained in the presence of F^-^ due to the low prevalence of dynamic traces. (**f**) Free energy diagram for RNA64 (orange) and EC64 (green) in the presence of 2 mM Mg^2+^. The relative free energies of the undocked and docked states were obtained from the dwell times in the docked and unfolded states, while the relative barrier heights were obtained using single-molecule transition state analysis (Methods).

It is well known that RNA folding is highly sensitive to many cell-specific factors including macromolecular crowding^28,29^, ionic conditions^30^ and proximal RNAP^23^. In particular, upon incorporation into a transcription complex containing DNA template and RNAP, the folding of the riboswitch could be altered by factors, such as macromolecular crowding, specific RNA-protein interactions and electrostatic interactions with the DNA template. To address this, we recorded smFRET traces with the riboswitch immobilized through biotinylated RNAP on PEG-passivated, streptavidin-coated quartz slides (Fig. 4a), which enabled observation of the riboswitch in an active, yet halted elongation complex (EC)^23,31,32^. Since our F^-^ riboswitch is derived from *Bacillus cereus*, we additionally performed transcription assays using RNA polymerase from the closely related species *Bacillus subtilis*, as well as the more distantly related *E. coli*. Both RNAPs exhibited pausing at U48 and fluoride-induced antitermination, although their behavior differed at locations further downstream than those investigated in this study (Supplementary Fig. 1). As a result, we elected to use the better studied *E. coli* RNAP in our experiments, which also allowed us to build on previous work^23^ that performed smFRET on ECs containing *E. coli* RNAP. While the two RNAPs may affect riboswitch behavior in somewhat different ways, we expect that the fundamental mechanism of ligand sensing that we have uncovered will be conserved as are the bacterial RNAPs.

Similar to the isolated RNA64, EC64 also shows three FRET states with E_FRET_ ≈ 0.40, 0.60 and 0.85. Both dynamic and static traces were observed as shown by off-diagonal and on-diagonal features in TODPs, respectively (Fig. 4). This suggests that in the presence of RNAP and the DNA template, the riboswitch retains the previously observed prefolded P, docked D and unfolded U states. For low- to mid-FRET transitions the observed rate constants are *k*_dock_ ≈ 3.5 s^−1^ and *k*_undock_ ≈ 0.5 s^−1^ in the presence of 2 mM Mg^2+^; note that the docking rate is ~3-fold higher than that observed for isolated RNA64 (Fig. 4e). Similar results were observed for high- to mid-FRET transitions, where the docking rate is increased by ~3.5-fold in EC64 (Supplementary Fig. 6). Moreover, we found that the SD conformation forms more readily for EC64 than RNA64. In the presence of 2 mM Mg^2+^, the population of the SD traces is 28% for EC64, which is ~threefold higher than that observed for RNA64 (Figs. 2a, 4b–d). Upon addition of 1 mM F^-^, the prevalence of SD traces further increased to 69%, or ~1.3-fold higher than that observed for RNA64 (Fig. 4d). We could not obtain reliable kinetic parameters for EC64 in the presence of F^-^ due to the dominance of SD states and thus the low prevalence of dynamic transitions. The increased number of SD traces in EC64 indicates that the transcription machinery thermodynamically stabilizes the docked conformation of the riboswitch (Fig. 4f). Still, the overall folding behavior observed for EC64 is the same as observed for RNA64 in the absence of RNAP, with the DD conformation predominant in the absence of F^-^ and the SD conformation becoming predominant in the presence of F^-^ (compare Figs. 2a and 4b–d).

To estimate the favorable effect of the transcription machinery on folding of the F^-^ riboswitch more quantitatively, we computed the free energy change upon docking and performed single-molecule transition-state analysis (smTSA, see Methods)^30,33–35^. In the presence of only 2 mM Mg^2+^, we determined that in EC64 the docked conformation is stabilized by ~0.61 kcal/mol compared to RNA64 (Fig. 4f), consistent with previous studies of molecular crowding^28,29^. Furthermore, smTSA revealed that the difference in the free energy of activation for docking in an EC relative to the isolated RNA, ${\triangle \triangle G}_{{dock}}^{++},$ is very similar at 0.58 kcal/mol at 2 mM Mg^2+^ (Fig. 4f), suggesting that the transition state is late with essentially all docking interactions already established, including those involving Mg^2+^ ions. Thus, we propose that the crowding near the surface of RNAP provides additional stabilizing interactions in the TS that accelerate the Mg^2+^-induced folding into the docked riboswitch conformation. (Of note, we did not perform TSA in the presence of F^-^ due to the small number of dynamic traces observed under those conditions).

### RNA polymerase plays a key role in the folding of riboswitch at early stages of transcription

**Fig. 5:**
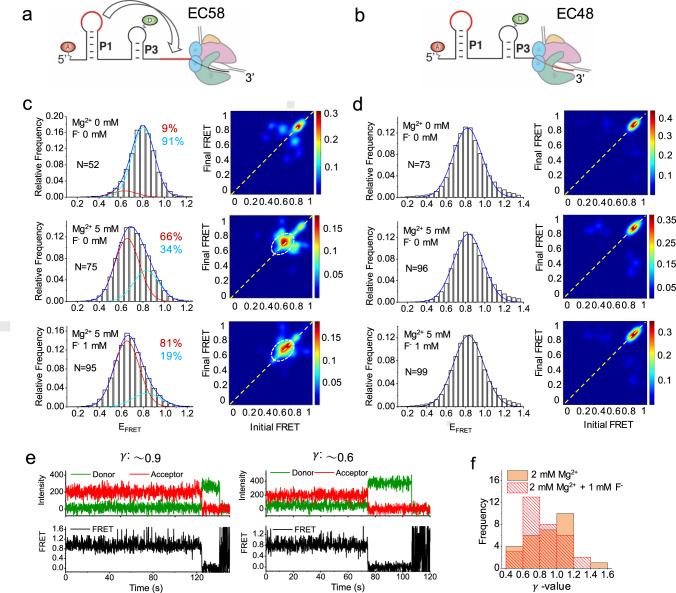
Effect of Mg^2+^ and F^-^ on EC58 and EC48. (**a, b**) Schematics of the fluoride riboswitch in an elongation complex at transcript lengths of 58 and 48 nucleotides, respectively. (**c, d**) FRET histograms and corresponding TODPs in the absence and presence of Mg^2+^ and F^-^ for EC58 and EC48, respectively. The FRET histograms are well fitted with two Gaussian peaks for EC58 shown in red and cyan for the mid- and high-FRET states, respectively, with the cumulative fit shown in blue; and a single Gaussian peak for EC48 shown in blue. The percent population of fitted peaks are shown in respective colors in each histogram panel, and the number of molecules that were analyzed is indicated by “N”. A dashed white circle highlights the presence of the SD conformation in TODPs for EC58. (**e**) Representative time traces for EC48 showing similar E_FRET_ values but different γ values, which were determined using fluorescence intensities of donor (*I*_*D*_) and acceptor (*I*_*A*_) before (pre) and after (post) photobleaching of the acceptor as ${{{{{\rm{\gamma }}}}}}=({{{{{{\rm{I}}}}}}}_{{{{{{\rm{A}}}}}},{{{{{\rm{Pre}}}}}}}-{{{{{{\rm{I}}}}}}}_{{{{{{\rm{A}}}}}},{{{{{\rm{Post}}}}}}})/({{{{{{\rm{I}}}}}}}_{{{{{{\rm{D}}}}}},{{{{{\rm{Post}}}}}}}-{{{{{{\rm{I}}}}}}}_{{{{{{\rm{D}}}}}},{{{{{\rm{Pre}}}}}}})$^38,39^. (**f**) Histogram of the ^γ^-parameter for EC48 in the absence and presence of F^-^ as indicated.

We next investigated the effects of the proximal RNAP on the folding of shorter riboswitch RNAs of lengths 58 and 48 (Fig. 5 and Supplementary Figs. 7, 8). For the isolated RNA58 as well as RNAP-containing EC58, the FRET histograms were best fitted with two Gaussian peaks representing high and mid-FRET states (Fig. 5d and Supplementary Figs. 7, 8). We again assigned the mid-FRET state to the docked conformation. For RNA58 almost all the traces were static in nature (Supplementary Fig. 7), while both dynamic and static traces were observed for EC58 (Fig. 5d). Of note, in EC58 we observed a shift from the P (high-FRET) state to the SD (static mid-FRET) state upon addition of Mg^2+^, with further addition of F^-^ stabilizing the SD state even more. This stands in contrast to EC64, in which Mg^2+^ and F^-^ induced a shift from the U state to the D state (Fig. 4).

We also performed smFRET experiments using an RNA variant, in complexes RNA58n and EC58n, in which the donor labeling position was altered (labeled at A39 instead of U33; Supplementary Note 1 and Supplementary Fig. 9). For this RNA, we disrupted the pseudoknot by two methods—by making a U45A/C46U double mutation, and by blocking the 5′ arm of the pseudoknot with an LNA oligonucleotide. In both cases, the population was shifted from the mid-FRET to the high-FRET state, indicating that the mid-FRET state here also represents a docked conformation that is depleted when pseudoknot formation is disfavored (Supplementary Fig. 10). Again, we observed that the F^-^ ligand favors the docked conformation in EC58n (Supplementary Note 1 and Supplementary Figs. 9–14). These results indicate that the transcription machinery facilitates folding of the riboswitch into the docked conformation, and that the P state is prevalent in the absence of Mg^2+^ and F^-^.

Interestingly, we observed very slow dynamics, on the time-scale of tens of seconds, for both the EC58 and EC58n (Supplementary Figs. 8 and 11). The intrinsic dynamics of pseudoknot formation is fast, as observed for EC64, in which the complete aptamer is accessible for folding. We hypothesized that the slow dynamics observed in EC58 and EC58n are caused by another parameter, possibly interaction of the riboswitch with RNAP. To test this, we performed further investigations including protein-induced fluorescence enhancement (PIFE)^36,37^ and single-molecule fluorescence lifetime measurements for the construct EC58n labeled with a single fluorophore at A39 (Supplementary Note 2 and Supplementary Fig. 14). We observed PIFE in both the absence and the presence of F^-^, indicating the existence of fluctuating RNA-RNAP interactions (Supplementary Fig. 14b). Furthermore, fluorescence lifetime measurements showed that addition of F^-^ causes the RNA to undergo a conformational change that increases the mobility of the fluorophore (Supplementary Fig. 14c–e). This change may result from the breaking of RNA-RNAP interactions that confine the fluorophore in the absence of F^-^.

In case of the shortest design EC48, representing a ligand-dependent transcription pause position (Supplementary Fig. 1), almost all of the population (>98%) remains in the high-FRET state in the absence and presence of Mg^2+^ and F^-^ (Fig. 5c). This can be rationalized given that EC48 cannot adopt the docked conformation because the 3′ segment of the aptamer is still sequestered within the RNA:DNA hybrid. In our in vitro transcription assay, we observed transcriptional pauses at even shorter RNA lengths (U30, U34, and U41; Supplementary Fig. 1) located in the aptamer domain that might extend the time available for the folding of the P1 hairpin. In agreement with the SHAPE-seq study^20^, these pauses are independent of the concentration of F^-^ (Supplementary Fig. 1 and Supplementary Note 3). By contrast, the ligand-dependent transcriptional pause at position U48 is proximal to the 3′ segment of the pseudoknot, and may provide time for the Mg^2+^ and F^-^ ligands to bind. Thus, the observed effect of F^-^ on the U48 pause half-life must occur through a local F^-^-induced change that is not detectable through the smFRET analysis presented so far.

**Fig. 6:**
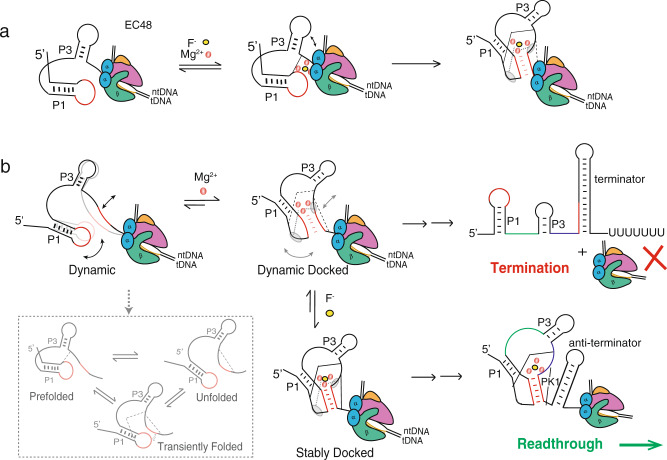
Models for ligand-dependent transcription regulation by the fluoride riboswitch. (**a**) A proposed local conformational change at early transcript length, where F^-^ binding pre-organizes the aptamer by poising the 5’ segment of the pseudoknot in close proximity to the RNAP exit channel, facilitating pseudoknot formation once the 3’ segment is transcribed. (**b**) If F^-^ binds late, the aptamer is highly dynamic in nature in the absence of Mg^2+^, transitioning among the prefolded, folded and unfolded states. The unfolded state is prevalent when the aptamer has been transcribed completely and the A40-U48 interaction can occur (RNA64). In the presence of physiological Mg^2+^ concentrations, the riboswitch dynamically samples a docked conformation similar to the ligand-bound conformation (“dynamic docked”). The binding of F^-^ stabilizes this conformation (to become “stably docked”), mediated by the linchpin long-range A40-U48 interaction.

To further investigate the possibility of a local F^-^-induced change in EC48, we measured gamma (γ) parameters from donor and acceptor time trajectories (Methods), where the deviation of the γ-value from a value of 1 is evidence of a change in the local environment around the fluorophore that could be due to interactions with protein or nucleic acids (Fig. 5e)^38,39^. For EC48, a broad distribution of γ-values was observed for individual molecules in the presence of 2 mM Mg^2+^, ranging from 0.4 to 1.6 with a maximum close to 1; upon addition of F^-^, the maximum population shifted to ~0.6 (Fig. 5f, time traces with different γ-values are shown in Fig. 5e). This deviation in γ supports the notion of an interaction of the L3 loop (where the donor fluorophore is located) with RNAP. The binding of F^-^ may bring L3 into closer proximity with the polymerase, facilitating the formation of the pseudoknot as additional RNA is transcribed (Fig. 6a).

## Discussion

We have explored in detail the conformational dynamics of the *B. cereus crcB* fluoride riboswitch at the single-molecule level. First, we investigated the folding dynamics of the aptamer and how it changes as a function of Mg^2+^ and F^-^ concentrations. On this foundation, we further explored the involvement of transcription machinery, specifically bacterial RNA polymerase, which induces the riboswitch to fold into the docked conformation, and explored other possible factors involved in the folding process. Using smFRET analysis, we resolved three conformational states: prefolded, unfolded, and docked, and determined their fractional populations and conformational dynamics including interconversion rates. We observed that the prefolded and unfolded states are prevalent before and after the aptamer has been fully transcribed, respectively, and each of these states is able to transition to the docked conformation in the presence of F^-^ ligand. Thus, the prefolded state may play a role as an intermediate on the pathway to the docked conformation at the early stages of transcription (Fig. 6). We also observed ligand-dependent switching behavior even at short transcript lengths, finding evidence that a local conformational change induced by F^-^ suppresses transcriptional pausing at U48.

The *B. cereus* fluoride riboswitch has previously been characterized by chemical-exchange saturation transfer (CEST) NMR spectroscopy^14^. In that study, highly localized fleeting dynamics were observed at the interface of the aptamer domain and the expression platform. The ligand-free aptamer was found to transiently access an excited conformational state and ligand binding allosterically suppressed that state, while the riboswitch adopted a tertiary structure in the presence of only Mg^2+^ similar to that in the presence of both Mg^2+^ and F^−^^14^. Using smFRET, we were able to identify key differences between the global behavior of the riboswitch in the presence of only Mg^2+^ and in the presence of Mg^2+^ and F^-^. Specifically, the DD state is prevalent in the presence of only Mg^2+^, getting “snap-locked” into the SD state upon F^-^ binding. The CEST-NMR study showed that the A40-U48 “linchpin” base-pair is important ﻿for aptamer folding and transcription regulation^14^. Here we observed that the A40-U48 base-pair plays a significant role in stabilizing the docked state in the presence of F^-^﻿, with a single mutation breaking this base-pair and completely abolishing the SD state. This stands in contrast to the pseudoknot-structured preQ_1_, ZTP and SAM II sensing riboswitches, where the docked conformation is mainly stabilized through the formation of the pseudoknot helix^5^. This distinction may be a unique consequence of the anionic F^-^ ligand binding to a polyanionic RNA.

Transcriptionally acting riboswitches are thought to be kinetically controlled and may be required to sense their ligands at the early stages of transcription in order to initiate the folding process before RNAP passes the intrinsic terminator hairpin^40^. SHAPE-seq indicated that the aptamer folds unstably in the absence of ligand, whereas in the presence of ligand it adopts a long-lived folded conformation^20^. This is consistent with our observation of a DD conformation in the presence of Mg^2+^ that transitions to an SD conformation upon addition of ligand. Given that each individual transcript makes its own “decision” between termination and read-through, single-molecule methods are particularly well-suited to analyze the key factors contributing to that decision. Considering the average transcription rate of 20-25 nt/sec in vivo^41^, there is a brief window of ~1-2 s for ligand binding between the departure of RNAP from the U48 pause and its arrival at the poly-U termination sequence pause. Of note, the prevalence of high stability SD traces (lifetime in the docked state at least 20 s) increases from 10% (RNA64) and 25% (EC64) without ligand to 50 and 70% with ligand, respectively. This indicates that a subpopulation of nascent RNAs will adopt a docked state at an early stage of transcription, and that they will persist in that state until the entire anti-terminator is formed. Additionally, the remaining (DD) population exhibits a dwell time in the docked state (τ_docked_) of ~1–2 s, meaning that termination or readthrough are both possible outcomes for the subpopulation of nascent RNAs that adopt this conformation. Thus, at least 70% of transcripts (the entire SD subpopulation + a fraction of the DD subpopulation) are expected to undergo read-through rather than termination in the presence of F^-^. This is consistent with previously reported in vitro transcription experiments that showed ~70–100% read-through depending on the rate of transcription^14^. Thus, the early binding of ligand during transcription would have a high impact on the downstream gene expression. Indeed, we observed that the γ-value of EC48 and the half-life of the pause at U48 both depend on F^-^ (Fig. 5 and Supplementary Fig. 1), suggesting that F^-^ may bind at an early stage of transcription, ﻿in contrast to the findings of the prior SHAPE-seq study^20^.

It is well known that Mg^2+^ promotes many riboswitches to fold into ﻿partially compact or docked-like conformations that become the basis for CS-mediated ligand binding^25,27,30^. A small change in the Mg^2+^ concentration may alter the nature of ligand-binding mechanism. For example, the preQ_1_ riboswitch from *Thermoanaerobacter tencongensis* follows an IF mechanism in the absence of Mg^2+^, while the addition of only 10 μM Mg^2+^ shifts to the mechanism to CS^30^. Similarly, small differences in aptamer sequence can significantly impact the ligand-mediated folding mechanism^27,42^. Here, we propose a mechanism (Fig. 6b) in which ligand binding by the aptamer domain follows a CS process, where physiological Mg^2+^ concentrations lead the riboswitch to dynamically sample a conformation similar to the F^-^-bound conformation (DD). F^-^ binding then stabilizes this conformation, leading to the observation of a long-lived docked state (SD).

The anionic ligand F^-^ does not directly interact with any specific nucleotide in the riboswitch but instead bridges a cluster of three Mg^2+^ ions in contact with the backbone phosphates near the PK1 helix^13^ (Fig. 6b). In contrast, riboswitches that sense cations, such as Mg^2+^ and Mn^2+^ bind their ligands largely through direct interactions with the phosphate backbone. Despite these differences, the Mn^2+^ riboswitch appears to follow a similar folding pathway as the one we uncovered for the F^-^ riboswitch. In the presence of Mg^2+^ alone, the Mn^2+^ riboswitch transiently samples a docked conformation that converts to a stably docked conformation upon ﻿addition of submillimolar Mn^2+^ ^25^. The similarity extends to the fact that a Mg^2+^ ion binds to the RNA very close to the riboswitch ligand, whereupon either Mn^2+^ or F^-^ binding can serve as a linchpin for the docking interaction.

Our results in elongation complexes with a proximal RNAP demonstrate that the transcription machinery facilitates folding of the riboswitch into the docked conformation (Fig. 4f). This behavior is analogous to that seen in a previous study, where RNAP was found to stabilize the docked pseudoknot conformation of the preQ_1_ riboswitch^23^, suggesting that stabilization of RNA folding by RNAP may be a more general phenomenon. In addition to molecular crowding, electrostatic interactions^23,43^ between the RNA and protein and slow solvation^34,44^ around proteins and RNAs may help lower the transition state energy as observed here (Fig. 4f).

Taken together, our findings provide an understanding of the ligand-sensing mechanism of the F^-^ riboswitch and the coupling between its conformational dynamics and the transcription machinery that may lay the basis for the development of novel antibiotics. We anticipate that the mechanism uncovered here may underlie the co-transcriptional folding pathways and regulatory functions of other riboswitches and RNAs.

## Methods

### Protein preparation

E. coli core RNAP containing an ‘AviTag’ biotinylation tag (GLNDIFEAQKIEWH) on the C terminus of the ß’ subunit was expressed from plasmid pIA1202 in BLR(DE3) cells^45^. The cells were grown at 37 °C in LB media supplemented with 100 µg/mL carbenicillin until OD_600_ reached around 0.52. They were induced with 0.4 mM IPTG and, at the same time, 20 µM biotin was introduced into the media. The cells were harvested 4 h postinduction. The protein was purified using a Ni-NTA agarose column, heparin column and finally a Mono Q ion-exchange column, followed by overnight dialysis into storage buffer (10 mM Tris-HCl, pH 7.5, 50% glycerol, 100 mM NaCl, 0.1 mM EDTA, 0.1 mM DTT) as described previously^23^ and stored at -80 °C.

### Preparation of RNA constructs for smFRET

Different lengths of riboswitch were prepared by splinted ligation of two (dye-labeled and nonlabeled) RNA oligonucleotides. Dye-labeled RNA oligonucleotides and nonlabeled RNA oligonucleotides bearing a 5′-phosphate were purchased from Dharmacon (GE Healthcare Life Sciences) and Integrated DNA Technologies, respectively. We used two different dye-labeled RNAs; the first contains a Cy5-9S label at U3 and a 5-aminohexylacrylamino-uridine (5-LC-N-U) at position U33, while other one contains a Dy547 label at the 2′ position of A39 and a 5′N3 at position U3; these constructs were labeled using a Cy3 and Cy5 mono-reactive dye pack (GE Healthcare Life Sciences), respectively. The RNA was isolated by ethanol precipitation.

For ligation, 300 pmol each of labeled RNA oligonucleotide and 5’phosphorylated RNA oligonucleotide were annealed to the complementary DNA or LNA splint (400 pmol) in a 20 µL of ligation buffer (150 mM NaCl, 10 mM Tris-HCl, pH 8.0, and 1 mM EDTA) by heating at 90 °C for 2 min and followed by cooling at 37 °C for 10 min and 22 °C for 10 min. This annealing mixture was diluted to 50 µL in T4 RNA ligase 2 buffer and 40 units of T4 RNA ligase 2 (New England Biolabs) were added. The reaction mixture was incubated at 37 °C for 3 h and the reaction was stopped by the addition of gel loading buffer (95% formamide, 1 mM EDTA). The RNA was purified by denaturing urea, 8–9% polyacrylamide gel electrophoresis (PAGE). The ligated RNA was electro-eluted from the gel (BioRad Model 422) and isolated by ethanol precipitation. After drying, the RNA pellet was dissolved in water and the concentration was determined using a NanoDrop spectrophotometer. During the labeling and ligation procedures, the samples having dye-labeled construct were protected from ambient light by aluminum foil.

### Sample preparation and smFRET experiments

We have performed smFRET experiments in the presence and absence of RNAP, where immobilization of sample is achieved using biotinylated RNAP and biotinylated capture probe (CP)-locked nucleic acids (LNA), respectively. The 3′ end of each riboswitch variant consisted of 10 nucleotides complementary to the template strand of the DNA transcription bubble or LNA sequence. For studying elongation complexes, we first prepared an artificial DNA bubble as previously described^23,46^. In brief, the sequence of nontemplate DNA (ntDNA) and template DNA (tDNA) strands (Table 2 S) were designed with 11 noncomplementary nucleotides in the middle of the bubble. Ten nucleotides of this noncomplementary region on the tDNA strand are complementary to those appended onto the 3′ end of RNA. The sequence of the CP was designed to be complementary to the same 10 nucleotides, and LNA bases were included to achieve a melting temperature T_m_ of more than 70 °C. The sequence of the 10 nucleotides that form the RNA:LNA or RNA:DNA hybrid was not based on the expression platform, and was identical in the RNA constructs of different lengths with the exception of three bases that had to be changed to accommodate the portion of the aptamer oligonucleotide that became part of the hybrid in the shortest design, RNA48.

For experiments in ECs, the RNA of interest was combined at a final concentration of 0.5 µM with tDNA and ntDNA each at 1 µM in imaging buffer (50 mM Tris-HCl, pH 7.5, 100 mM KCl). The mixture was annealed by incubating at 90 °C for 2 min, then 37 °C for 10 min and then RT for 10 min. This annealing mixture was diluted in imaging buffer supplemented with 1 mM MgCl_2_ to a final concentration of 50 nM and 0.37–0.42 µM *E. coli* RNAP was added; this mixture was incubated for 15 min at 37 °C and then kept on ice. This reaction mixture was diluted in imaging buffer to a final concentration of 100–200 pM RNA in and flowed into the imaging chamber (which had previously been passivated with a 10:1 ratio of PEG to biotinylated PEG, then incubated for 10–15 min with a 0.2 mg/mL solution of streptavidin). For experiments on isolated RNA, 0.5 µM RNA was combined with 5–7.5 µM of CP-LNA in the imaging buffer. The mixture was annealed by incubating at 90 °C for 2 min, then 37 °C for 10 min and then RT for 10 min. The annealing mixture was diluted in imaging buffer to a concentration of 20–100 pM RNA and flowed onto the imaging chamber. After incubation for 2-10 min, unbound RNA molecules were removed by extensive flushing of the imaging chamber with imaging buffer.

Unless otherwise noted, all experiments were carried out in 50 mM Tris-HCl, pH 7.5, 100 mM KCl, supplemented with varying concentrations of MgCl_2_ and fluoride as indicated in the text and figures. An enzymatic oxygen scavenging system (OSS) was also included consisting of 165 U/mL glucose oxidase from *Aspergillus niger*, 2170 U/mL catalase from *Corynebacterium glutamicum*, 44 mM glucose and 5 mM trolox to minimize photo-bleaching and photo-blinking of dye molecules^47^. This oxygen scavenger was chosen based on previously reported measurements of RNAP elongation activity in buffers containing various OSS components^23^. The imaging chamber was incubated with each buffer for 10 min prior to data collection. All smFRET movies were recorded at room temperature (22 °C) on a prism-based TIRF microscope using a CCD (I-Pentamax, Princeton Instruments) or sCMOS camera (Hamamatsu ORCA-Flash4.0 V3) with the exposure time of 50 ms, unless otherwise specified. A continuous laser source of 532 nm was used to excite Cy3 or DY547 and the emission from both Cy3/DY547 (donor) and Cy5 (acceptor) were recorded simultaneously on the same camera. The sample was directly excited using a 640 nm laser at the beginning and the end of each movie to verify the presence of Cy5. Labview was used for all the commands and data acquisition.

### smFRET data analysis

All of the smFRET data analysis was performed using custom Matlab scripts (The Math Works). For each dataset, donor-acceptor pairs were identified and localized, and the intensity of each fluorophore was tracked throughout the movie. For further analysis, we manually selected the traces that adhere to the following criteria: Single-step photobleaching, a total (donor + acceptor) fluorescence intensity of >250 (arbitrary units), a fluorescence duration of at least 100 frames (prior to photobleaching) and, if transitions were observed, anticorrelation between donor and acceptor signals. These selection criteria ensure that the traces that get selected originate from single donor-acceptor pairs, not from aggregates or background impurities. All selected traces were processed to subtract background and correct for cross-talk and fluorescence bleed-through. The FRET efficiency (E_FRET_) was calculated by using background-corrected fluorescence intensities of donor (I_D_) and acceptor (I_A_) and the equation ${E}_{{FRET}}={I}_{A}/({I}_{A}+{I}_{D})$. FRET histograms were created by sorting the normalizing E_FRET_ and taking the same weightage from each trace to avoid the population inequality due to the different time length of traces that occurs due to photobleaching. OriginLab was used for plotting and fitting of histograms. The approximate distances (r) between the fluorophores were calculated using the mean FRET (E) values and an R_0_ value of 54 Å for the Cy3–Cy5 the dye pair using the following equation^48^:

1 $$E={R}_{0}^{6}/({R}_{0}^{6}+{r}^{6})$$

### HMM and kinetic analysis

For kinetic analysis, the QuB software was used as described previously^24,49^, where the traces were idealized using hidden Markov modeling (HMM) with a three-state model corresponding to two undocked states (low and high FRET) and a docked state (mid-FRET) using the segmental k-means algorithm. The idealized smFRET traces were further analyzed using custom Matlab scripts that identified transitions, created TODPs and exported dwell-times. TODPs are heat maps that show the fraction of traces that exhibit a given type of transition at least once^24^, with all traces weighted equally. In brief, regardless of the number of transitions in a dynamic trace, only a single transition is counted to construct the TOPD, which avoids the over-representation of molecules exhibiting many fast transitions. A typical TODP includes both dynamic traces (fast transitions along with slow or rare transitions) and static traces. The sub-populations of different FRET transitions from dynamic traces show up as off-diagonal contours in the TODP heat map, whereas on-diagonal contours represent the sub-population of static traces. For the dynamic traces, dwell times of each state were extracted and converted to cumulative dwell-time histograms. Lifetimes in the undocked (τ_prefolded_ and τ_unfolded_) and docked (τ_docked_) states were obtained by fitting with multi-exponential functions in OriginPro 8.5. Rate constants of docking and undocking were then calculated as *k*_dock_ = 1/τ_unfolded_ or 1/τ_prefolded_ and *k*_undock_ = 1/τ_docked_.

Since we observed two distinct population of traces, static docked as well as dynamic docked, the equilibrium constant for docking (*K*_*dock*_) was estimated from both types of traces, which was further used to calculate an overall docking Gibbs free energy as follows:

2 $${K}_{{dock}}=\frac{{k}_{{dock}}}{a{k}_{{undock}}+\left(1-a\right){k}_{{photobleach}}}$$

3 $${\triangle G}_{{dock}}^{0,{RNA}64}=-{RT}{ln}{K}_{{dock}}\left({RNA}64\right)$$

4 $${\triangle} {G}_{dock}^{0,EC64}=-{RT}{ln}{K}_{dock}(EC64)$$

where *a* and *(1-a)* are the fractional populations of dynamic and static traces, respectively. Furthermore, the free energies of docking ${\left(\right.\triangle G}_{{dock}}^{0}$) observed for RNA64 and EC64 were used to compute the change in the equilibrium free energy ${\triangle} {\triangle} {G_{dock}}^{0}$of folding:

5 $${\triangle \triangle G}_{{dock}}^{0}={\triangle G}_{{dock}}^{0,{RNA}64}-{\triangle G}_{{dock}}^{0,{EC}64}$$

Note: Here we assumed a lower limit of *K*_*dock*_, which is limited by the photobleaching rate of static docked traces. However, in case of dynamics traces, the cumulative dwell-time of docked state (τ_docked_) was best fitted with three-exponential function having a slowest time constant of around 20 s (with 2–3% population), which is very close to the average time constant (19–24 s) for the static docked traces. Thus, we assume the observed *K*_*dock*_ would be very close to the actual value. Furthermore, we compute the free energy change in presence of only 2 mM Mg^2+^ (for both RNA64 and EC64), and not in presence of F^-^ due to the small number of dynamic traces and more number of static docked traces, which may contribute to high error.

### Single-molecule transition-state analysis (TSA)

For RNA64 and EC64, single-molecule TSA was carried out as previously described^30,34^. The rate constants of docking *k*_dock_ observed for RNA64 and EC64 were used to compute the changes in the free energy barrier for docking (${\triangle \triangle G}_{{dock}}^{++}$) induced by RNAP, as follows:

7 $${\triangle \triangle G}_{{dock}}^{++}=-{RT}{ln}({k}_{{dock}}^{{RNA}64}/{k}_{{dock}}^{{EC}64})$$

Although TSA has been extensively used to study the folding of single-domain proteins, it can also be applied to investigate bimolecular reactions where folding is coupled to ligand binding^35^. Recently, a similar approach has been used to study the folding of RNA, using “mutation” of a ligand to alter its interactions with the RNA during its folding^30,33–35^. In the current study, we measured ${\triangle \triangle G}_{{dock}}^{++}$ by treating the isolated riboswitch (RNA64) as the “mutant”, given that RNAP contacts are absent in this form.

### Calculation of Gamma values

We measured the Gamma parameter (γ) for traces that show similar E_FRET_, but have differences between the donor and acceptor time traces in terms of their quantum yield^39^. γ is determined from the change in the intensities of donor and acceptor upon photobleaching of the acceptor as follows^38^:

8 $${{{{{\rm{\gamma }}}}}}=({{{{{{\rm{I}}}}}}}_{{{{{{\rm{A}}}}}},{{{{{\rm{Pre}}}}}}}-{{{{{{\rm{I}}}}}}}_{{{{{{\rm{A}}}}}},{{{{{\rm{Post}}}}}}})/({{{{{{\rm{I}}}}}}}_{{{{{{\rm{D}}}}}},{{{{{\rm{Post}}}}}}}-{{{{{{\rm{I}}}}}}}_{{{{{{\rm{D}}}}}},{{{{{\rm{Pre}}}}}}})$$

where I_A or D_ is the fluorescence intensity of the donor or acceptor fluorophore, and *Pre* and *Post* subscripts indicate intensities before and after photobleaching of the acceptor, respectively.

### Excited-state fluorescence lifetime of DY547 of single riboswitch

Fluorescence lifetimes were recorded using a fluorescence lifetime imaging (FLIM) system (Alpha 5, ISS Inc., Urbana-Champaign, IL). The sample was excited at 532 nm, selected by AOFT from a white light excitation source (Fianium WL-SC-400-8-PP) using a 582/75 nm emission filter. First, a laser scanning confocal image was recorded, then the excitation light was focused on a specific molecule and the fluorescence intensity recorded overtime to generate a time-dependent fluorescence decay. Individual decays were fitted using deconvolution of the instrument response function (IRF), yielding the fluorescence lifetimes of single riboswitches.

### Single-round Transcription Assays

#### DNA templates

A 199-nucleotides DNA template including the fluoride riboswitch from *B. cereus* under the control of the T7A1 promoter was cloned into pUC19 plasmid between EcoRI and BamHI restriction sites. In addition, 25 nucleotides not found in the wild-type sequence were inserted after the promoter sequence in order to generate a 25-nucleotide stretch in which the RNA transcript lacks any uracil residues (EC-25) except for the +2 position dependent of the ApU dinucleotide used to initiate the transcription. Transcription templates for in vitro transcription were generated by PCR using the ‘T7A1-PCR’ forward DNA oligonucleotide and the ‘crcB-reverse’ reverse DNA oligonucleotide. (Supplementary Table 2). For *B. subtilis* transcription, the T7A1 promoter was replaced by the LambdaPr promoter using forward DNA oligonucleotide ‘LambdaPr-CrcB-FWD’ (Supplementary Table 2).

#### In vitro Transcription Assay

Halted complexes (EC-25) were prepared in transcription buffer (20 mM Tris-HCl, pH 8.0, 20 mM NaCl, 20 mM MgCl_2_, 14 mM 2-mercaptoethanol, 0.1 mM EDTA) containing 25 µM ATP/CTP mix, 50 nM α^32^P-GTP (3000 Ci/mmol), 10 µM ApU dinucleotide primer (Trilink), and 50 nM DNA template. *E. coli* RNAP holoenzyme (New England Biolabs) was added to 100 nM, and the mixture was incubated for 10 min at 37 °C. In the case of *B. subtilis* transcription core RNAP and SigA factor were incubated for 30 min at 30 °C with a 1:4 ratio (RNAP:SigA) to reconstitute the holoenzyme prior to the transcription reaction. The sample was passed through a G50 column to remove any free nucleotides. To complete the transcription reaction all four rNTPs were added concomitantly with heparin (450 µg/mL) to prevent the re-initiation of transcription. Time pausing experiments were performed using 10 µM rNTPs. The mixture was incubated at 37 °C, and reaction aliquots were quenched at the desired times into an equal volume of loading buffer (95% formamide, 1 mM EDTA, 0.1% SDS, 0.2% bromophenol blue, 0.2% xylene cyanol). Sequencing ladders were prepared by combining the halted complex with a chase solution containing 250 µM of each rNTP, in addition to one 3’-OMethyl rNTP (at 5 µM for 3’-OMe GTP and 2 µM for 3’-OMe ATP, UTP and CTP). Reaction aliquots were denatured before loading 5-8 µL of each onto a denaturing 8 M urea, 6% polyacrylamide sequencing gel. The gel was dried and exposed to a phosphor screen (typically overnight), which was then scanned on a Typhoon Phosphor Imager (GE Healthcare Life Sciences).

The half-life of transcriptional pausing was determined by calculating the fraction of each RNA pause species compared to the total amount of RNA for each time point, which was analyzed with pseudo-first-order kinetics to extract the half-life^50^. For each determination we subtracted the background signal. Error bars in transcription quantification represent the standard deviation of the mean from at least two independent replicates.

### Reporting summary

Further information on research design is available in the Nature Research Reporting Summary linked to this article.

## Supplementary information

Supplementary Information

Reporting Summary

## Data Availability

The data supporting the findings of this study are available from the corresponding authors upon reasonable request. The source data underlying Figs. 2c, d, e, and 4d, e, Supplementary Fig. 1c, Supplementary Fig. 4, and Supplementary Fig. 6 are provided in a Source Data file and in Supplementary Table 1. Study data have been deposited in the University of Michigan Deep Blue Data repository (10.7302/pa6y-fb55). Source data are provided with this paper.

## Source data

Source Data

## Code availability

Custom scripts used to analyze the smFRET have been deposited in the University of Michigan Deep Blue Data repository (10.7302/pa6y-fb55).

## References

[1] Sherwood AV, Henkin TM (2016). Riboswitch-Mediated Gene Regulation: Novel RNA Architectures Dictate Gene Expression Responses. Annu. Rev. Microbiol..

[2] Mandal M, Breaker RR (2004). Gene regulation by riboswitches. Nat. Rev. Mol. Cell Biol..

[3] Serganov A, Nudler E (2013). A Decade of Riboswitches. Cell.

[4] Mironov AS (2002). Sensing Small Molecules by Nascent RNA: A Mechanism to Control Transcription in Bacteria. Cell.

[5] Jones CP, Ferré-D’Amaré AR (2017). Long-Range Interactions in Riboswitch Control of Gene Expression. Annu. Rev. Biophys..

[6] Frieda KL, Block SM (2012). Direct Observation of Cotranscriptional Folding in an Adenine Riboswitch. Sci. (80-.).

[7] Greenlee EB (2018). Challenges of ligand identification for the second wave of orphan riboswitch candidates. RNA Biol..

[8] Sherlock ME, Breaker RR (2020). Former orphan riboswitches reveal unexplored areas of bacterial metabolism, signaling, and gene control processes. RNA.

[9] Tang D-J (2020). A SAM-I riboswitch with the ability to sense and respond to uncharged initiator tRNA. Nat. Commun..

[10] Deigan KE, Ferré-D’Amaré AR (2011). Riboswitches: Discovery of Drugs That Target Bacterial Gene-Regulatory RNAs. Acc. Chem. Res..

[11] Baker, J. L. et al. Widespread Genetic Switches and Toxicity Resistance Proteins for Fluoride. *Science*. **335**, 233–235 (2012). 10.1126/science.1215063 PMC4140402 22194412

[12] Li S (2013). Eukaryotic resistance to fluoride toxicity mediated by a widespread family of fluoride export proteins. Proc. Natl Acad. Sci. USA..

[13] Ren A, Rajashankar KR, Patel DJ (2012). Fluoride ion encapsulation by Mg2+ ions and phosphates in a fluoride riboswitch. Nature.

[14] Zhao B, Guffy SL, Williams B, Zhang Q (2017). An excited state underlies gene regulation of a transcriptional riboswitch. Nat. Chem. Biol..

[15] Wickiser JK, Winkler WC, Breaker RR, Crothers DM (2005). The Speed of RNA Transcription and Metabolite Binding Kinetics Operate an FMN Riboswitch. Mol. Cell.

[16] Ray S, Chauvier A, Walter NG (2019). Kinetics coming into focus: single-molecule microscopy of riboswitch dynamics. RNA Biol..

[17] Uhm H, Kang W, Ha KS, Kang C, Hohng S (2018). Single-molecule FRET studies on the cotranscriptional folding of a thiamine pyrophosphate riboswitch. Proc. Natl Acad. Sci. USA..

[18] Hua B, Panja S, Wang Y, Woodson SA, Ha T (2018). Mimicking Co-Transcriptional RNA Folding Using a Superhelicase. J. Am. Chem. Soc..

[19] Hua B (2020). Real-time monitoring of single ZTP riboswitches reveals a complex and kinetically controlled decision landscape. Nat. Commun..

[20] Watters KE, Strobel EJ, Yu AM, Lis JT, Lucks JB (2016). Cotranscriptional folding of a riboswitch at nucleotide resolution. Nat. Struct. Mol. Biol..

[21] Strobel EJ, Cheng L, Berman KE, Carlson PD, Lucks JB (2019). A ligand-gated strand displacement mechanism for ZTP riboswitch transcription control. Nat. Chem. Biol..

[22] Watters KE, Yu AM, Strobel EJ, Settle AH, Lucks JB (2016). Characterizing RNA structures in vitro and in vivo with selective 2′-hydroxyl acylation analyzed by primer extension sequencing (SHAPE-Seq). Methods.

[23] Widom JR (2018). Ligand Modulates Cross-Coupling between Riboswitch Folding and Transcriptional Pausing. Mol. Cell.

[24] Blanco, M. & Walter, N. G. Analysis of Complex Single-Molecule FRET Time Trajectories. in *Methods in Enzymology* **472**, 153–178 (Academic Press, 2010). 10.1016/S0076-6879(10)72011-5 PMC3012381 20580964

[25] Suddala KC (2019). Local-to-global signal transduction at the core of a Mn2+ sensing riboswitch. Nat. Commun..

[26] Weikl TR, Paul F (2014). Conformational selection in protein binding and function. Protein Sci..

[27] Haller A, Soulière MF, Micura R (2011). The Dynamic Nature of RNA as Key to Understanding Riboswitch Mechanisms. Acc. Chem. Res..

[28] Daher M, Widom JR, Tay W, Walter NG (2018). Soft Interactions with Model Crowders and Non-canonical Interactions with Cellular Proteins Stabilize RNA Folding. J. Mol. Biol..

[29] Dupuis NF, Holmstrom ED, Nesbitt DJ (2014). Molecular-crowding effects on single-molecule RNA folding/unfolding thermodynamics and kinetics. Proc. Natl Acad. Sci. USA..

[30] Suddala KC, Wang J, Hou Q, Walter NG (2015). Mg2+ Shifts Ligand-Mediated Folding of a Riboswitch from Induced-Fit to Conformational Selection. J. Am. Chem. Soc..

[31] Daube S, von Hippel P (1992). Functional transcription elongation complexes from synthetic RNA-DNA bubble duplexes. Sci. (80-.).

[32] Sidorenkov I, Komissarova N, Kashlev M (1998). Crucial Role of the RNA:DNA Hybrid in the Processivity of Transcription. Mol. Cell.

[33] Silverman SK, Cech TR (2001). An early transition state for folding of the P4-P6 RNA domain. RNA.

[34] Bokinsky G (2003). Single-molecule transition-state analysis of RNA folding. Proc. Natl Acad. Sci..

[35] Fersht AR, Daggett V (2002). Protein Folding and Unfolding at Atomic Resolution. Cell.

[36] Hwang H, Myong S (2014). Protein induced fluorescence enhancement (PIFE) for probing protein-nucleic acid interactions. Chem. Soc. Rev..

[37] Hwang H, Kim H, Myong S (2011). Protein induced fluorescence enhancement as a single molecule assay with short distance sensitivity. Proc. Natl Acad. Sci. USA..

[38] Ray S, Bandaria JN, Qureshi MH, Yildiz A, Balci H (2014). G-quadruplex formation in telomeres enhances POT1/TPP1 protection against RPA binding. Proc. Natl Acad. Sci. USA.

[39] Dahan M (1999). Ratiometric measurement and identification of single diffusing molecules. Chem. Phys..

[40] Garst AD, Batey RT (2009). A switch in time: detailing the life of a riboswitch. Biochim. Biophys. Acta.

[41] Vogel U, Jensen KF (1994). The RNA chain elongation rate in Escherichia coli depends on the growth rate. J. Bacteriol..

[42] Suddala KC (2013). Single transcriptional and translational preQ1 riboswitches adopt similar pre-folded ensembles that follow distinct folding pathways into the same ligand-bound structure. Nucleic Acids Res..

[43] Gupta A, Gribskov M (2011). The Role of RNA Sequence and Structure in RNA–Protein Interactions. J. Mol. Biol..

[44] Yadav R, Sengupta B, Sen P (2016). Effect of sucrose on chemically and thermally induced unfolding of domain-I of human serum albumin: Solvation dynamics and fluorescence anisotropy study. Biophys. Chem..

[45] Kay BK, Thai S, Volgina VV (2009). High-throughput Biotinylation of Proteins. Methods Mol. Biol..

[46] Kolb KE, Hein PP, Landick R (2014). Antisense Oligonucleotide-stimulated Transcriptional Pausing Reveals RNA Exit Channel Specificity of RNA Polymerase and Mechanistic Contributions of NusA and RfaH. J. Biol. Chem..

[47] Aitken CE, Marshall RA, Puglisi JD (2008). An Oxygen Scavenging System for Improvement of Dye Stability in Single-Molecule Fluorescence Experiments. Biophys. J..

[48] Ishii Y, Yoshida T, Funatsu T, Wazawa T, Yanagida T (1999). Fluorescence resonance energy transfer between single fluorophores attached to a coiled-coil protein in aqueous solution. Chem. Phys..

[49] Qin F, Li L (2004). Model-Based Fitting of Single-Channel Dwell-Time Distributions. Biophys. J..

[50] Landick R, Wang D, Chan CL (1996). Quantitative analysis of transcriptional pausing by Escherichia coli RNA polymerase: his leader pause site as paradigm. Methods Enzymol..

