# Supplementary material for: An anionic ligand snap-locks a long-range interaction in a magnesium-folded riboswitch
Source: Nat Commun. 2022 Jan 11;13:207. doi: 10.1038/s41467-021-27827-y (PMC8752731; doi:10.1038/s41467-021-27827-y)
Supplement: Supplementary file 1 — Supplementary Information [file 41467_2021_27827_MOESM1_ESM.pdf]

Supplementary Information:

An anionic ligand snap-locks a long-range interaction in a magnesium-folded riboswitch

Rajeev Yadav[1,2], Julia R Widom[1,3], Adrien Chauvier[1] & Nils G Walter[1,*]

[1]Single Molecule Analysis Group, Department of Chemistry and Center for RNA Biomedicine, University of Michigan, Ann Arbor, MI 48109, USA

[2]Present address: Department of Physics and Astronomy, Michigan State University, East Lansing, MI 48824, USA

[3]Present address: Department of Chemistry and Biochemistry, University of Oregon, Eugene, OR 97403, USA

*e-mail: nwalter@umich.edu

Supplementary Note 1: Experiments with an alternative donor labeling position

To validate our findings, we further performed smFRET experiments at RNA lengths of 48 and 58 with a different donor labeling position (Supplementary Fig. 9a). In this design, the donor (DY547) is placed at A39, rather than U33 as in the previous construct; the acceptor position remains the same at the 5'-end. When referring to this new design, we place "n" after the number designating a particular RNA species (EC58n and RNA58n, for example).

For the isolated RNA58n, we observed only one FRET state and no changes upon addition of Mg^{2+} and F^- (Supplementary Fig. 9b,d). The FRET histograms were best fitted with a single Gaussian peak centered around $E_{FRET} \approx 0.9$ in the absence and presence of Mg^{2+} and F^- (Supplementary Fig. 9d). In contrast, the elongation complex EC58n exhibits an additional mid-FRET state in the absence of Mg^{2+} and F^-, which becomes more prominent in the presence of Mg^{2+} and F^- (Supplementary Fig. 9c,e). Since F^- favors the docked pseudoknot conformation of the riboswitch, the additional peak in EC58n presumably results from pseudoknot formation in the presence of RNAP. To ensure that the mid-FRET state represents the docked conformation in this new design, we performed control experiments in which the pseudoknot was disrupted either by mutation (U45A/C46U)[1] or with the L1 block sequence (Supplementary Fig. 10). Both of these alterations depress the population of the mid-FRET state and abolish its response to Mg^{2+} and F^-, confirming that the mid-FRET state represents the docked conformation.

Having noticed that the dynamics are very slow at this transcript length for constructs with the original labeling scheme, we performed smFRET measurements with an exposure time of 400 ms. For EC58n, the FRET histograms were best fitted with two Gaussian peaks centered around $E_{FRET} \approx 0.55$ and 0.9 and no effect was observed upon addition of F^- in the absence of Mg^{2+} (Supplementary Fig. 11c). In the presence of 5 mM Mg^{2+}, the population of the mid-FRET state

was 20%, which increased to 45% in the presence of 0.5 mM F^-. These results are in agreement with those shown in the main text for the corresponding RNA designs with the original labeling scheme (Fig. 4), indicating that F^- binds only in the presence of Mg^{2+} and induces folding into the docked conformation.

We observed both dynamic and static traces as shown by diagonal and off-diagonal features in TODPs, respectively (Supplementary Fig. 11c,d). In the absence of Mg^{2+}, a static population is observed in the high FRET sate, which on addition of F^- remains almost unchanged. However, in the presence of Mg^{2+} F^- addition leads to a decrease in the static high-FRET population and a concomitant increase in the dynamic population, as well as a small static population in the mid-FRET state at high F^- concentration (Supplementary Fig. 11d). We observed very slow dynamics for this RNA design (Supplementary Fig. 11e), where the rate constants are observed as $k_{\mathrm{dock}} \approx 0.06\ \mathrm{s}^{-1}$ and $k_{\mathrm{undock}} \approx 0.062\ \mathrm{s}^{-1}$ in the absence of Mg^{2+} and almost no change was observed on addition of F^-. In the presence of 5 mM Mg^{2+}, the rates are observed as $k_{\mathrm{dock}} \approx 0.03\ \mathrm{s}^{-1}$ $k_{\mathrm{undock}} \approx 0.032\ \mathrm{s}^{-1}$, which on addition of 0.1 mM F^- changes to $k_{\mathrm{dock}} \approx 0.04\ \mathrm{s}^{-1}$ $k_{\mathrm{undock}} \approx 0.025\ \mathrm{s}^{-1}$. All these results support that the docked state is stabilized in the presence of F^-.

Because the intrinsic dynamics of the aptamer are fast (discussed in the main text for RNA64), the comparably slow dynamics of EC58n point towards involvement of other factors. Additionally, the mid-FRET (docked) state was observed in EC58n but not RNA58n (Supplementary Fig. 9). These additional factors could include interactions of the riboswitch with either the whole transcription machinery or with only the DNA template or RNAP. To differentiate between these possibilities, we performed smFRET experiment on RNA58n in the presence of only the DNA template, where the riboswitch was immobilized through a biotinylated non-template DNA (ntDNA) strand. Under these conditions, we observed only the high-FRET state

(Supplementary Fig. 12), similar to what was seen for isolated RNA58n (Supplementary Fig. 9d). This clearly indicates that the docked state observed in EC58n appears due to the presence of RNAP; potential interactions of the riboswitch with RNAP are discussed in the main text and the following section.

We observed mainly the high-FRET state for isolated RNA48n as well as EC48n and did not observe any detectable change upon addition of Mg^{2+} and F^- (Supplementary Fig. 13). Based on these results and those discussed in the main text for RNA48 and EC48, we conclude that this state is prevalent before the riboswitch has been fully transcribed, and we term it the precursor state.

Supplementary Note 2: Interaction of the riboswitch with RNAP at transcript length 58

We first tested the hypothesis that the riboswitch interacts with RNAP by performing protein induced fluorescence enhancement (PIFE)[2,3] measurements on EC58n, labeled with only a donor fluorophore at A39 (Supplementary Fig. 14a). The fluorescence intensity varied over time in most traces, in both the absence and presence of F^- (Supplementary Fig. 14b), indicating that the local environment of the donor dye is changing (a restricted environment leads to fluorescence enhancement). This could only be possible if there is a structural change in the riboswitch that changes the extent to which the region in the vicinity of the fluorophore (possibly the L3 loop) is interacting with RNAP. As discussed in the main text, this effect was also seen for EC48.

Furthermore, we used confocal fluorescence microscopy to record the fluorescence lifetime of the donor at the single-molecule level (Supplementary Fig. 14c,d). For this experiment, we immobilized the riboswitch (EC58n with only Dy547) through biotinylated *E. coli* RNAP on a PEG-passivated, streptavidin coated cover glass. A laser scanning confocal image was recorded

using a pulsed laser, which was then focused onto a specific molecule. Time-dependent fluorescence decays were then collected and fitted by deconvolution of the instrument response function (IRF), yielding the fluorescence lifetimes of single riboswitches. The fluorescence lifetime distributions in the presence of 2 mM Mg^{2+} and 2 mM Mg^{2+} plus 1 mM F^- are shown in Supplementary Fig. 14e. In the presence of only Mg^{2+}, the distribution was best fitted with a single Gaussian peak centered around 1.8 ns, and in the presence of Mg^{2+} and F^- was best fitted with two peaks centered around 1.3 ns and 1.8 ns. We conclude that the longer lifetime observed in the absence of F^- results from confinement of the fluorophore as shown schematically in Supplementary Fig. 14a. When the riboswitch converts into the docked conformation upon addition of F^-, the fluorophore becomes free to move and exhibits a shorter lifetime as a result.

Supplementary Note 3: *In vitro* transcription assays

Overall, our single-molecule results indicate that stabilizing interactions within an EC facilitate F^--and Mg^{2+}-dependent folding of the nascent fluoride riboswitch. During RNA synthesis, pausing by RNAP plays a critical role in guiding the folding of RNAs within a short time window.[4,5] We used single-round transcription assays to probe how pausing by RNAP impacts the folding pathway of the riboswitch and ligand-mediated regulation of transcription. In our *in vitro* transcription assay, we observed transcriptional pauses at various locations, some of them (U30, U34 and U41) located in the aptamer domain. In agreement with a prior SHAPE-seq study,[1] these pauses are independent of the concentration of F^- (Supplementary Fig. 1). They extend the time available for the folding of the P1 hairpin. Two pauses (U48 and U72) are observed in the expression platform. The U72 pause is located downstream of the terminator hairpin and is not detected in the presence of ligand. It could therefore act as a decision point where

pausing by RNAP allows time for F^- to bind; upon further addition of 5 nucleotides by transcription finally the terminator hairpin nucleates[1].

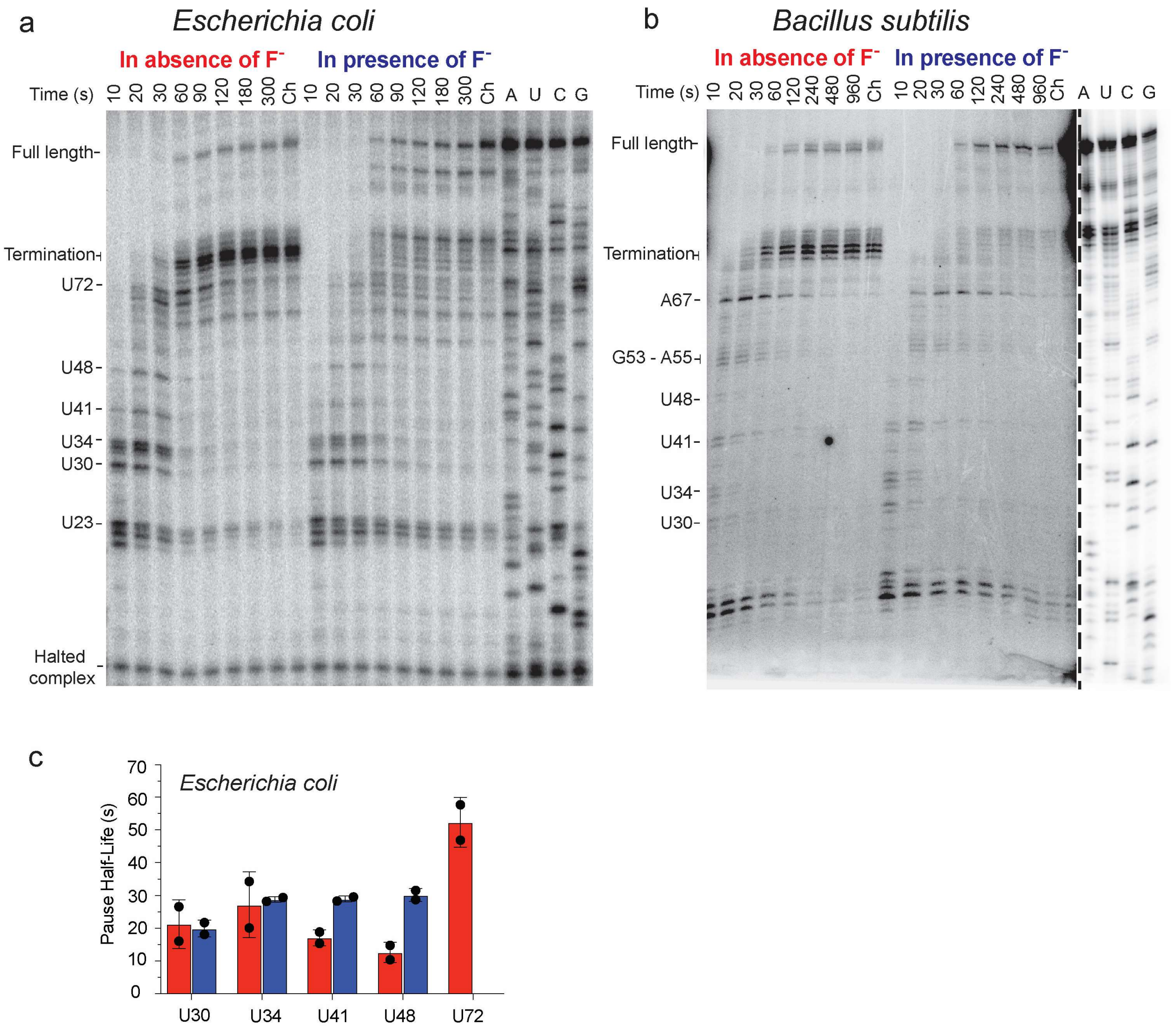

Supplementary Figure 1. *In vitro* transcriptional pausing during synthesis of the fluoride riboswitch. (a, b) Representative denaturing urea-acrylamide gel with RNA polymerase from *Escherichia coli* (*E. coli*) and *Bacillus subtilis* (*B. subtilis*), respectively. The pausing pattern is different with *B. subtilis* compare to *E. coli* transcription. The pauses at U30, U34, U41, U48 are common for both the polymerase, while the pause at U72 is specific to *E. coli* and the pauses at G53, U54, A55 and A67 are specific for *B. subtilis*. Both *In vitro* transcription assays were repeated independently for at least two times and got similar results in both cases. (c) Pause half-life with *E. coli* transcription in the absence (red) and presence (blue) of F^- as indicated. Error bars are presented as ± standard deviation for n = 2 independent experiments.

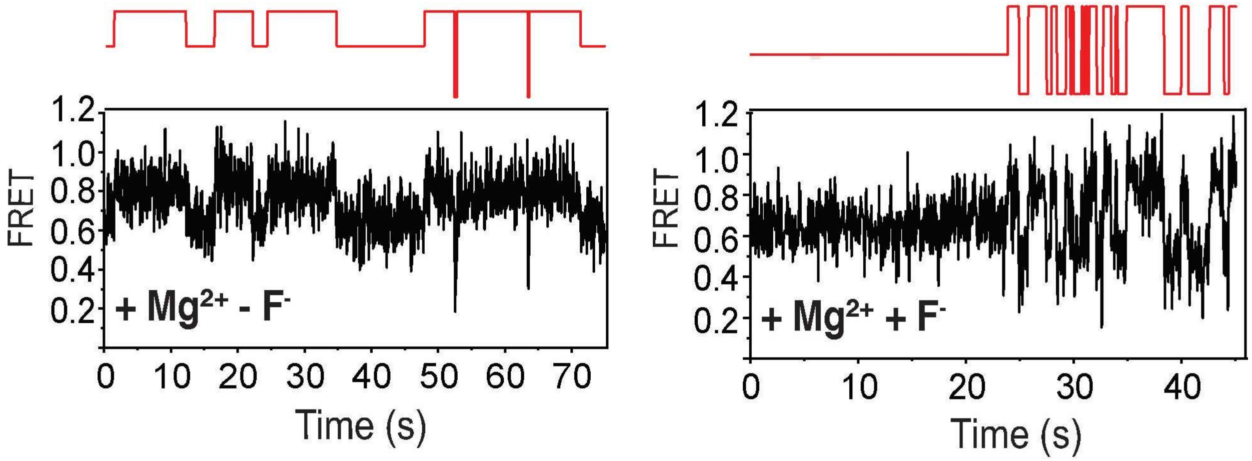

Supplementary Figure 2. Examples of time traces for RNA64 exhibiting all three FRET states. Examples of smFRET traces that show transitions between all three FRET states for RNA64 are observed in both the absence and presence of Mg^{2+} and F^- (observed only 5-10% of total traces in both the absence and presence of F^-). The left trace is in presence of Mg^{2+}only, while the right trace is in the presence of both Mg^{2+}*and* F^-. The HMM fit (red line) for each trace shows the three FRET states.

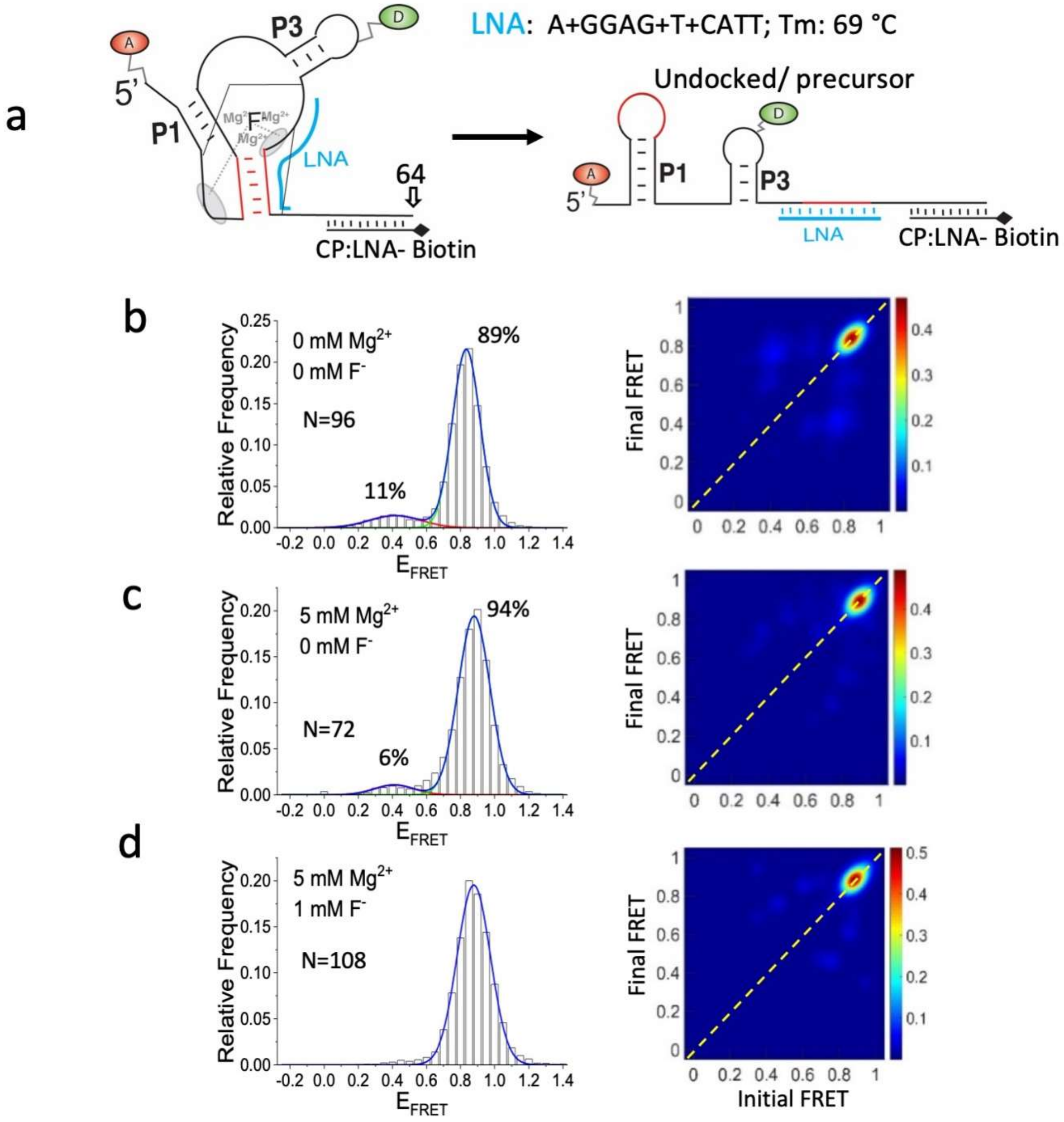

Supplementary Figure 3. smFRET data on RNA64 with LNA sequence complementary to A39-U48. This LNA sequence, which has three nucleotides (+G, +T, +C) modified with a 2'-O, 4'-C methylene bridge on their ribose, blocks pseudoknot formation along with A40-U48 long-range interactions. (a) Structure of the riboswitch with the LNA sequence shown in cyan. (b-d) FRET histograms and their corresponding TODPs (b) in the absence of Mg^{2+} and F^-, (c) in the presence of 5 mM Mg^{2+} only and (c) in the presence of 5 mM Mg^{2+} and 1 mM F^-.

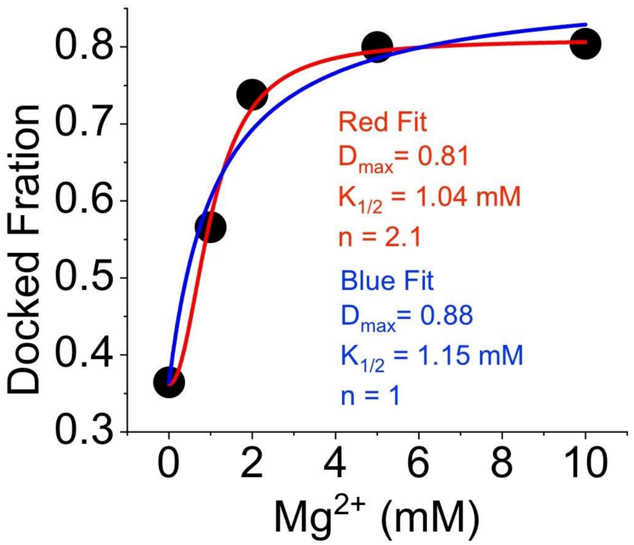

Supplementary Figure 4. The population of the docked state changes with Mg^{2+} concentration. This titration curve is well fitted by a Hill equation with a cooperativity coefficient of 2.1 (red); the fit is poor when using a non-cooperative model (blue).

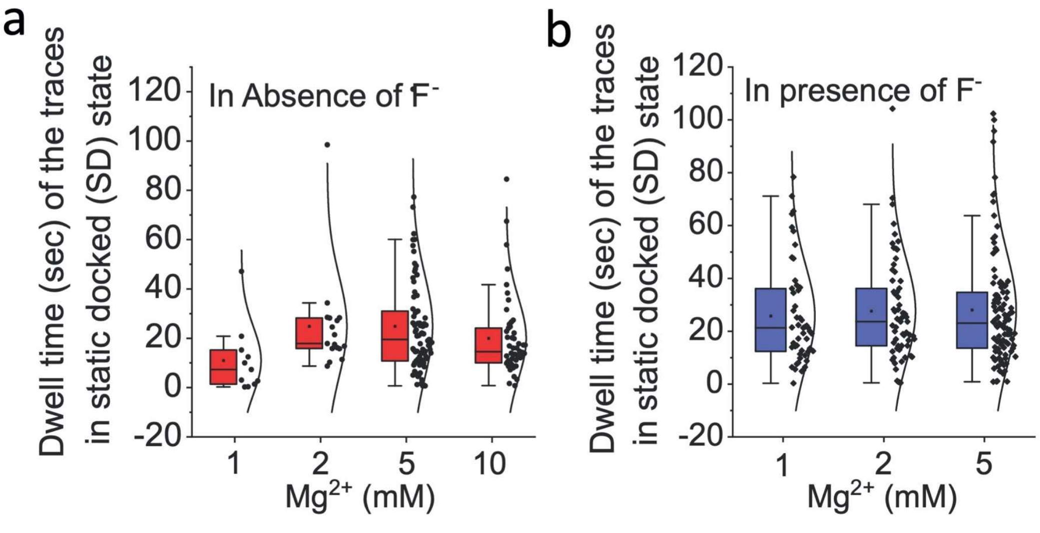

Supplementary Figure 5. Photobleaching times of static traces in the mid-FRET state of RNA64 (SD traces). Data are shown at different concentrations of Mg^{2+} (a) in the absence of F^- and (b) in the presence of 1 mM F^-. The mean dwell times with the interquartile range (IQR) are 11 s (IQR: 1.7 s - 12 s, n = 11) , 25 s (IQR: 16 s - 27 s, n = 17), 25 s (IQR: 11 s - 29 s, n = 79) and 20 s (IQR: 10 s - 24 s, n = 50) in the presence of 1, 2, 5 and 10 mM Mg^{2+} (without F^-), and 26 s (IQR: 12 s - 36 s, n = 51), 28 s (IQR: 15 s - 35 s, n = 65), 28 s (IQR: 14 s - 35 s, n = 107) in the presence of 1, 2 and 5 mM Mg^{2+} (with 1 mM F^-). These data indicate that the molecules remain in the SD conformation longer than the mean photobleaching time of around 20 s.

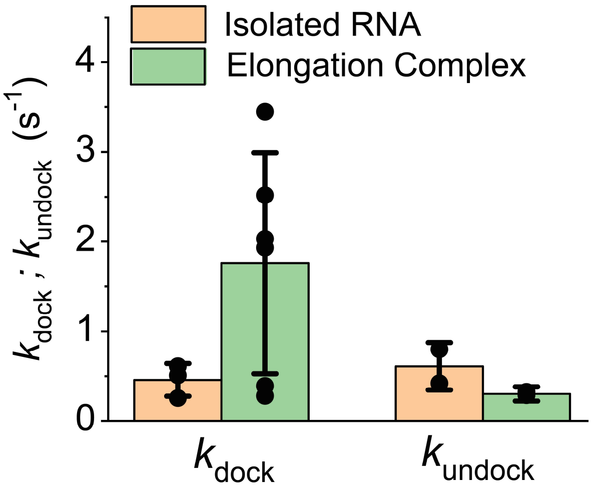

Supplementary Figure 6. Proximity of RNA polymerase favors the docked conformation of RNA64. Kinetic parameters for high- to mid-FRET (prefolded to docked state) transitions. The errors represent the standard deviation of two separate datasets. Due to the small number of dynamic traces, bootstrapping was used to calculate error bars for the EC data and is presented as ± standard deviation for n ≥ 2 independent data sets (with the values k_{dock}: 0.5 ± 0.2 s^{-1} and k_{undock}: 0.6 ± 0.3 s^{-1} for the isolated RNA and k_{dock}: 1.7 ± 1.2 s^{-1} and k_{undock}: 0.3 ± 0.1 s^{-1} for the Elongation Complex).

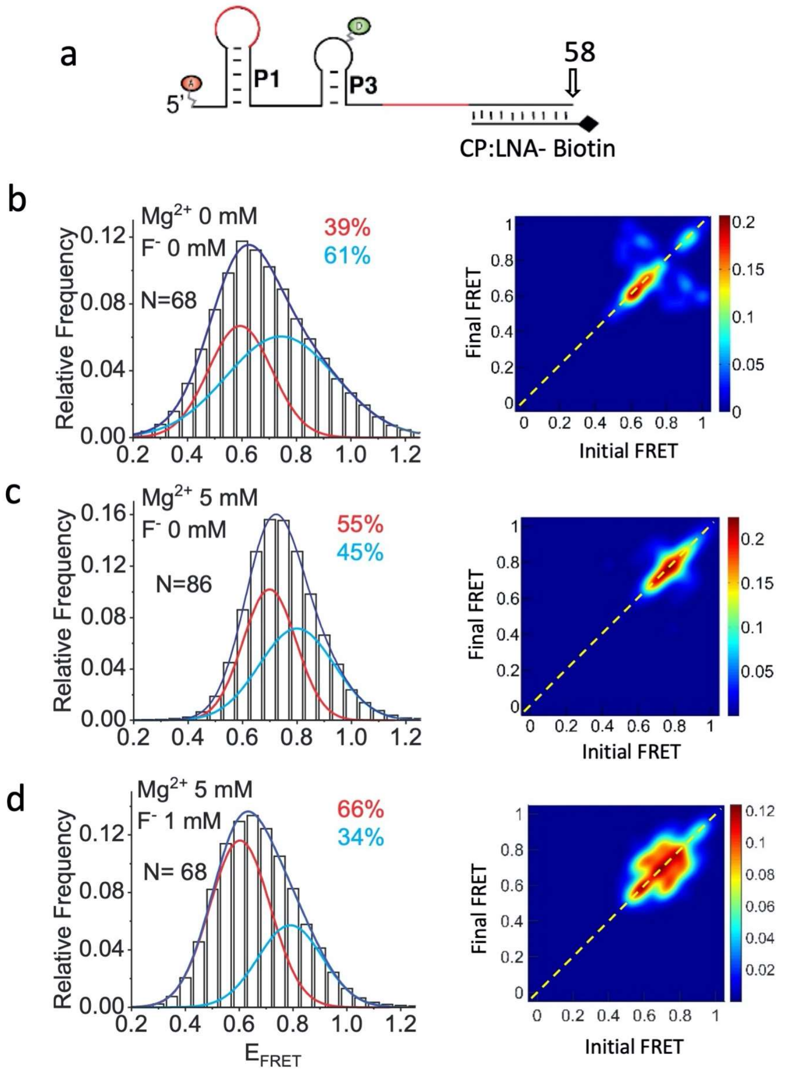

Supplementary Figure 7. **smFRET histograms for RNA58**. (a) A schematic representation of RNA58. (b-d) smFRET histograms and their corresponding TODPs in the absence and presence of Mg^{2+} and F^- as indicated on each plot. The percent population of both fitted peaks are shown in respective colors in each histogram panel, and the number of molecules that were analyzed is indicated by "N".

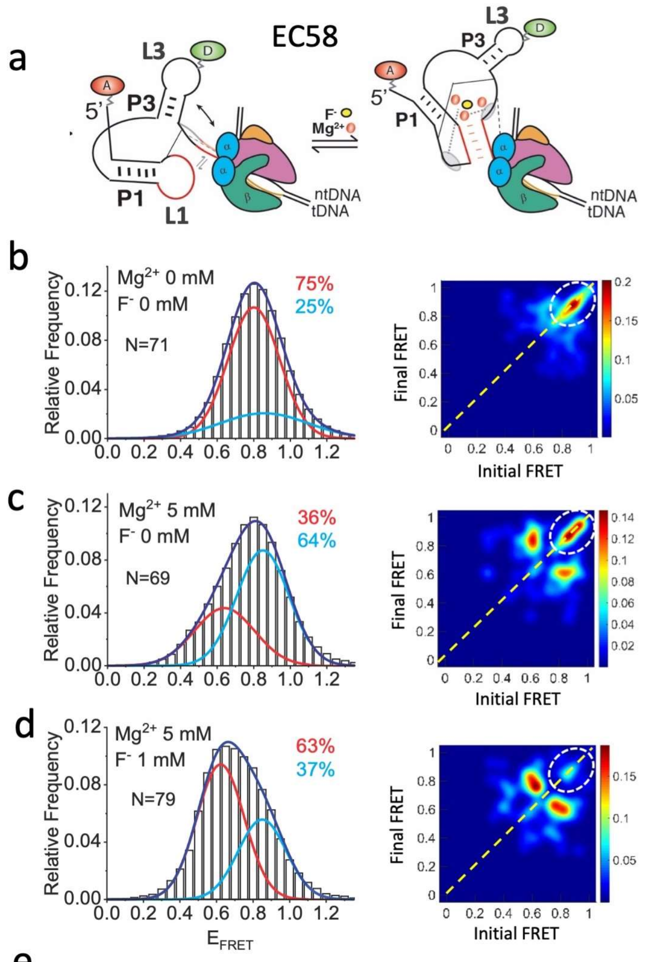

e

	k_{undock} (s^{-1})	k_{dock} (s^{-1})
+Mg^{2+} - F^-	0.068 ± 0.013	0.035 ± 0.010
+Mg^{2+} + F^-	0.065 ± 0.001	0.058 ± 0.002

Supplementary Figure 8. **Dynamics for EC58**. (a) Schematic of precursor (left) and docked (right) RNA structures for EC58. (b-d) FRET histograms and TODPs for EC58, recorded at an exposure time of 400 ms. In the absence of Mg^{2+} and F^-, most of the population is in the high-FRET (precursor) state; on addition of Mg^{2+} and F^- transitions occur between the high- and mid-FRET states. The population of the mid-FRET state increases on addition Mg^{2+} and F^- due to replacement of high-FRET static traces (highlighted by on-diagonal white circles) with dynamic traces as shown in the TODPs. The percent population of both fitted peaks are shown in respective colors, and the number of molecules that were analyzed is indicated by "N" in each FRET histogram panel. (e) Kinetic parameters for transitions between the high- and mid-FRET states. The reported errors are the standard error of the fit.

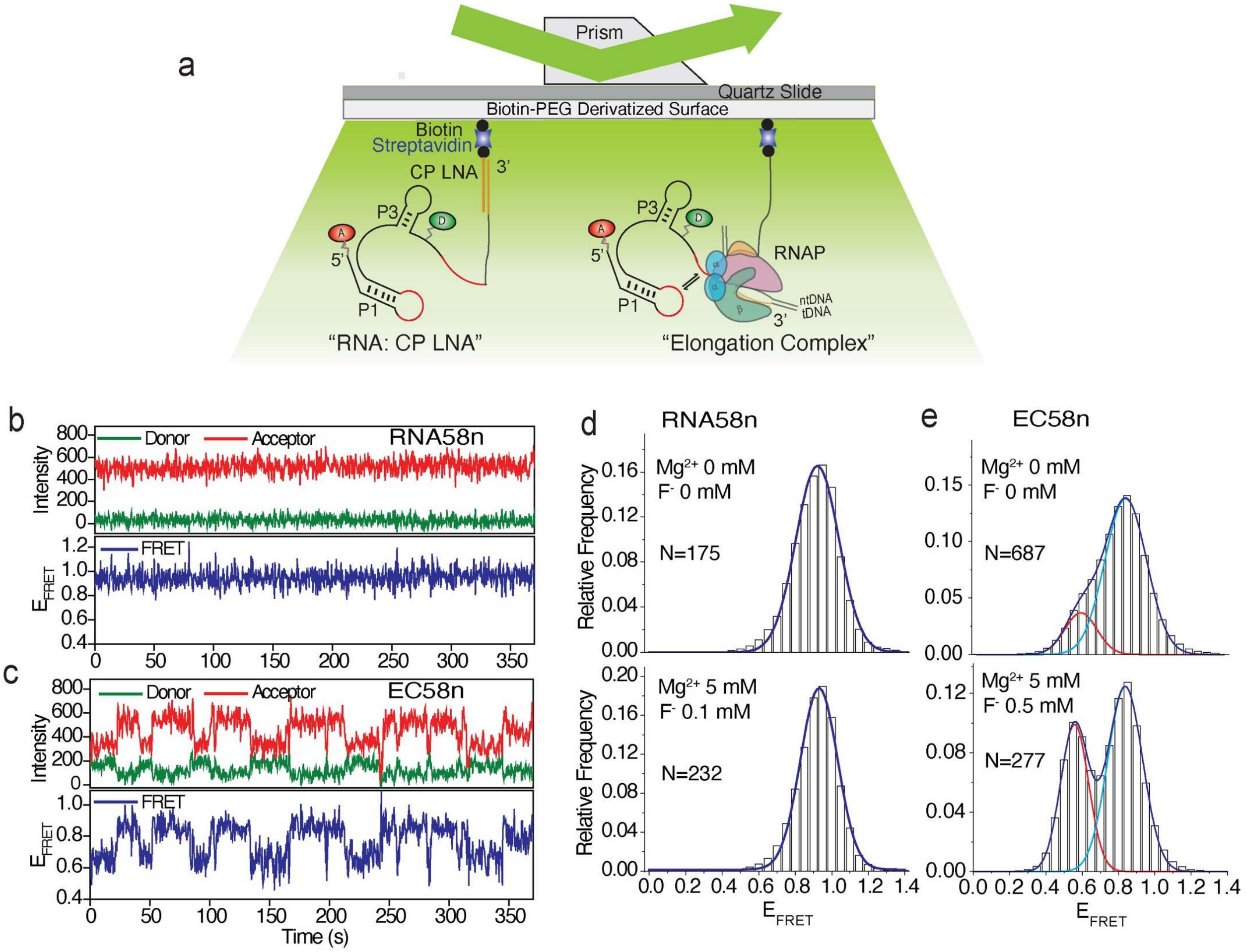

Supplementary Figure 9. RNA58n and EC58n with donor fluorophore at A39. (a) smFRET experimental setup for isolated RNA and elongation complex (note the new donor labeling position). The RNA is immobilized through biotinylated CP LNA or biotinylated *E. coli* RNAP, as indicated. (b, c) Representative FRET time traces for RNA58n and EC58n in presence of 5mM Mg^{2+}, respectively. (d, e) FRET distribution observed for RNA58n (d) and EC58n (e), in the absence and presence of Mg^{2+} and F^{-}. Only the high-FRET state is observed for RNA58n, while for EC58n a mid-FRET state is also observed. The number of molecules that were analyzed is indicated by "N" in the FRET histogram panel.

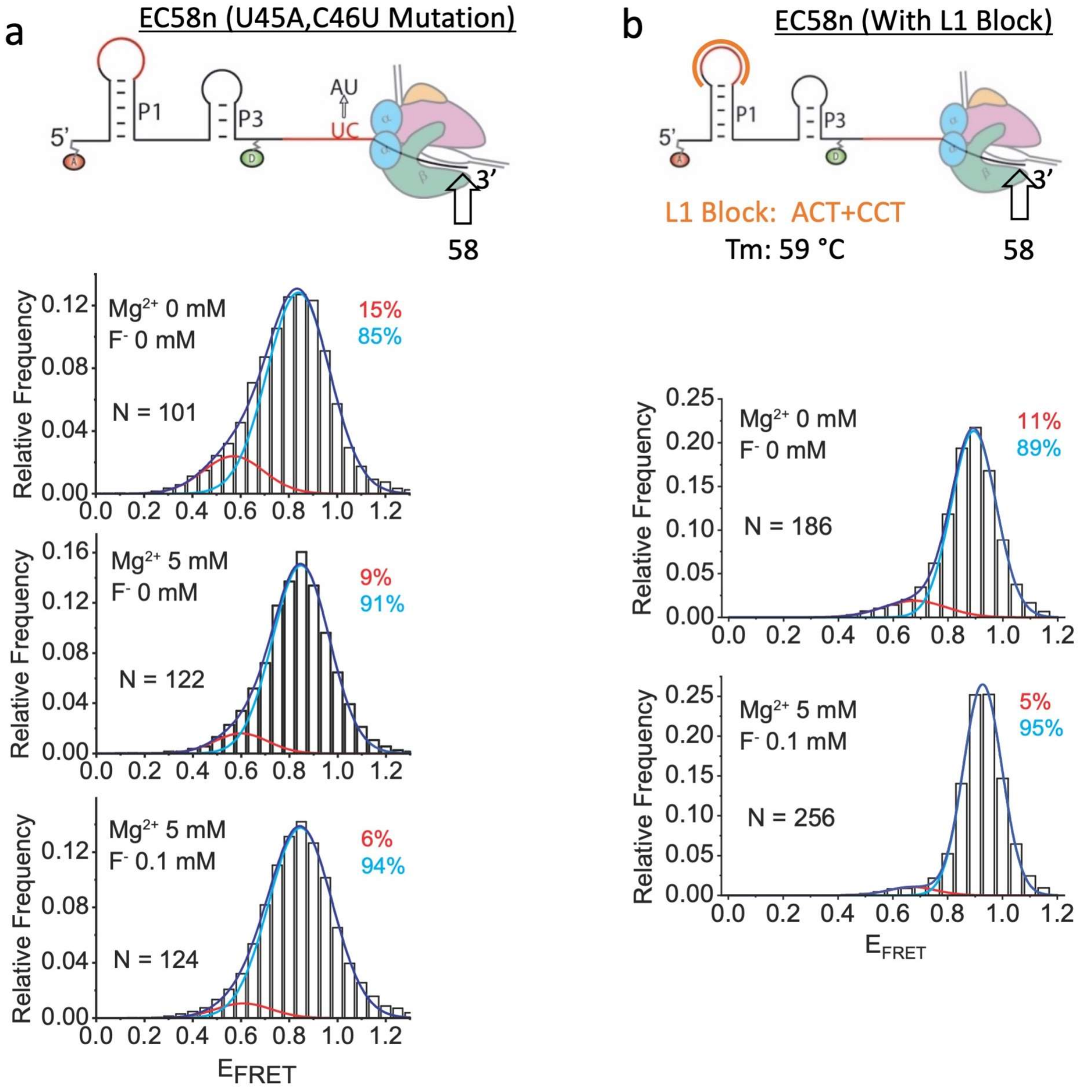

Supplementary Figure 10. The mid-FRET state in EC58n represents the docked conformation. smFRET measurements for EC58n (a) with a U45A,C46U double mutation and (b) with L1 block sequence, both of which disrupt pseudoknot formation. In both cases the population of the mid-FRET state is reduced relative to the wild-type and without the L1 block sequence. The number of molecules that were analyzed is indicated by "N" in the FRET histogram panel.

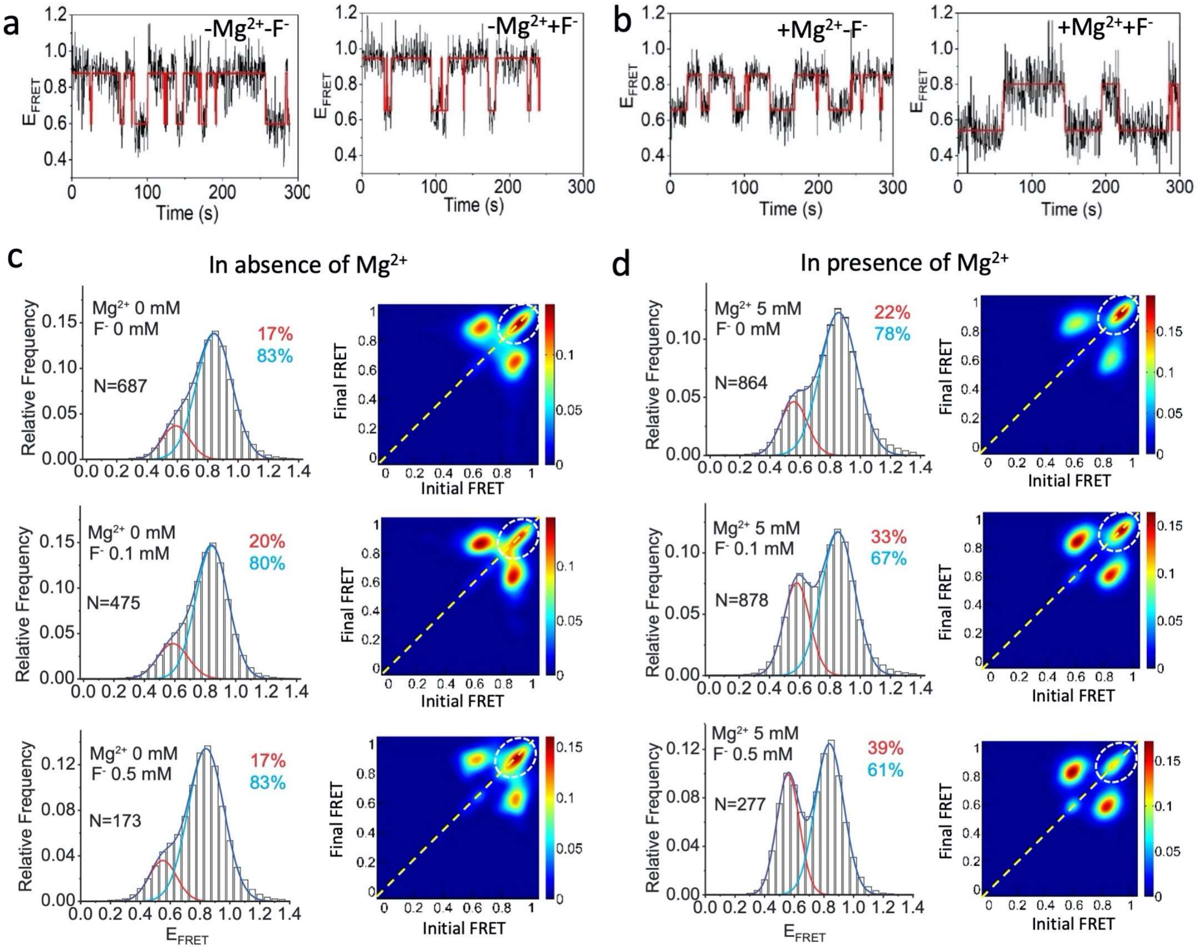

e

	k_{undock} (s^{-1})	k_{dock} (s^{-1})
- Mg^{2+} - F^-	0.062 ± 0.015	0.06 ± 0.014
+ Mg^{2+} - F^-	0.032 ± 0.004	0.03 ± 0.005
+ Mg^{2+} + F^-	0.025 ± 0.004	0.04 ± 0.01

Supplementary Figure 11. **Dynamics for EC58n.** (a, b) Representative FRET efficiency traces for EC58n in the absence (a) and presence (b) of Mg^{2+} at an exposure time of 400 ms. (c, d) smFRET histograms and their corresponding TODPs at varying concentrations of F^- in the absence (c) and presence (d) of Mg^{2+}. In both cases FRET histograms were best fitted with two Gaussian peaks. The percent population of both fitted peaks are shown in respective colors in each histogram panel. In the presence of Mg^{2+}, the population of the mid-FRET state increases on addition of F^- due to replacement of high-FRET static traces (highlighted by on-diagonal white circles) with dynamic traces as shown in the TODPs in panel d. The number of molecules that were analyzed is indicated by "N" in the FRET histogram panel. (e) Kinetic parameters for interconversion between the mid- and high-FRET states. The reported errors are the standard deviation of at least three independent datasets.

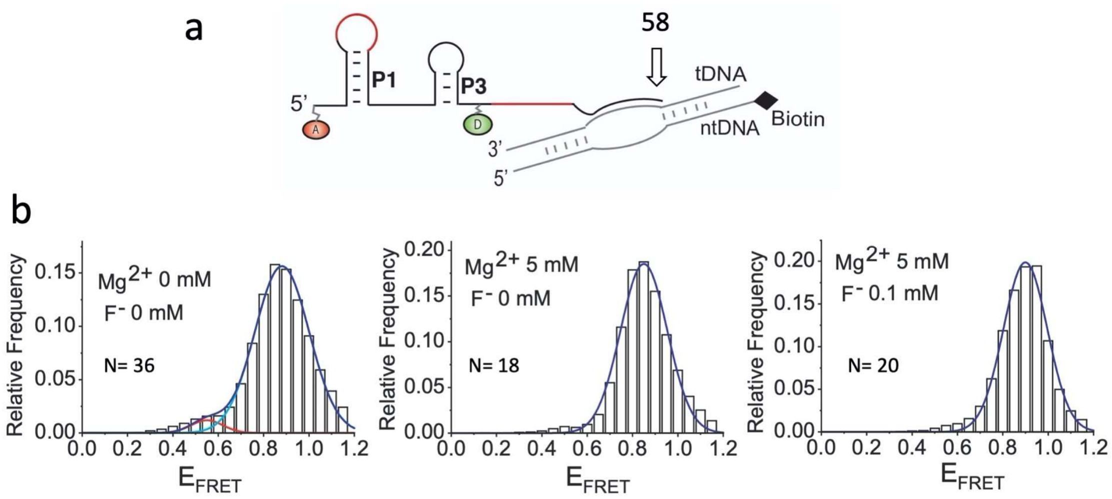

Supplementary Figure 12. RNA58n with DNA template. This control experiment suggests that the mid-FRET peak (around $E_{FRET} \approx 0.55$) observed in EC58n requires the presence of RNAP, as it is not observed when only the DNA template is present. (a) Schematic of RNA58n with DNA template experiment (b) smFRET histograms observed in the absence and presence of Mg^{2+} and F^{-}.

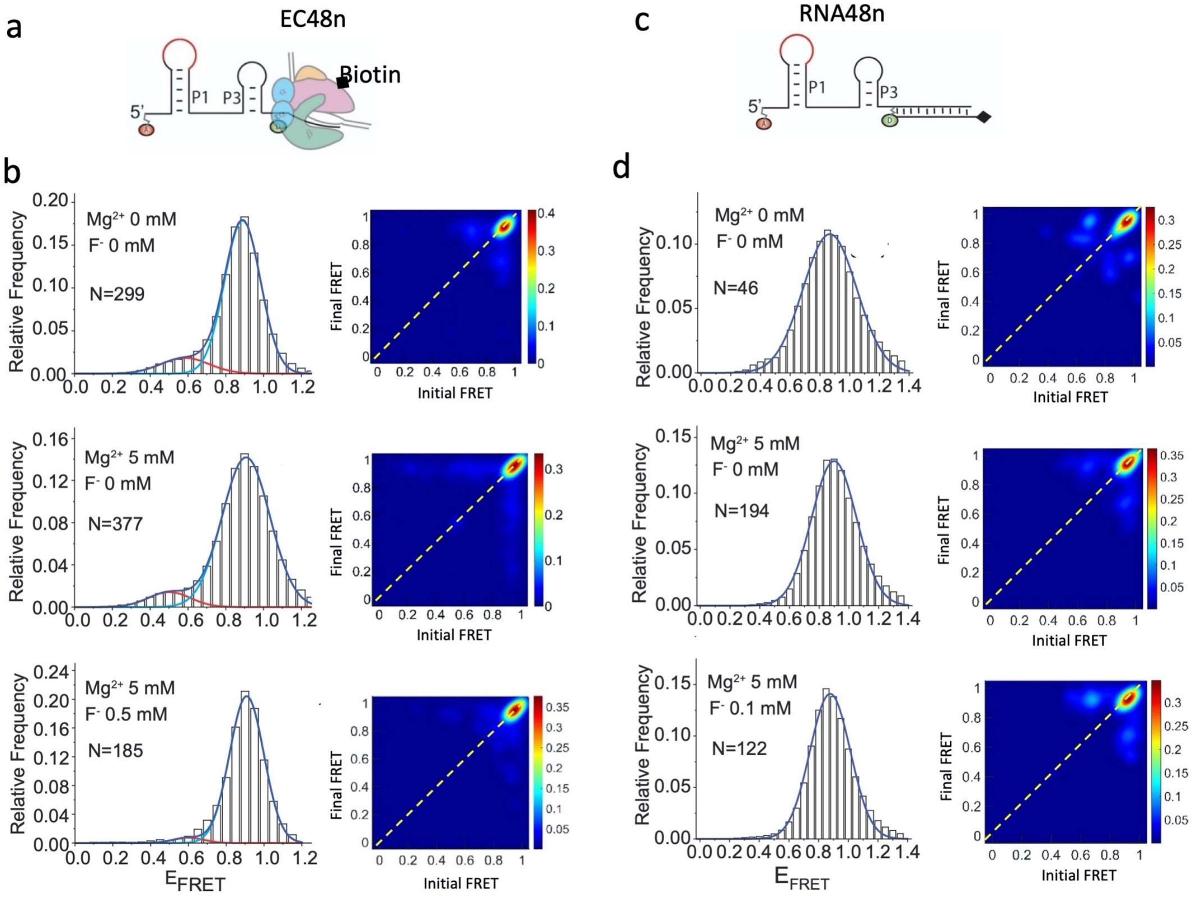

Supplementary Figure 13. smFRET data for EC48n and RNA48n. (a) Schematic of EC48n. (b) smFRET histograms and their corresponding TODPs for EC48n in the absence and presence of Mg^{2+} and F^-. (c) Schematic of RNA48n. (d) smFRET histograms and their corresponding TODPs for RNA48n in the absence and presence of Mg^{2+} and F^-.

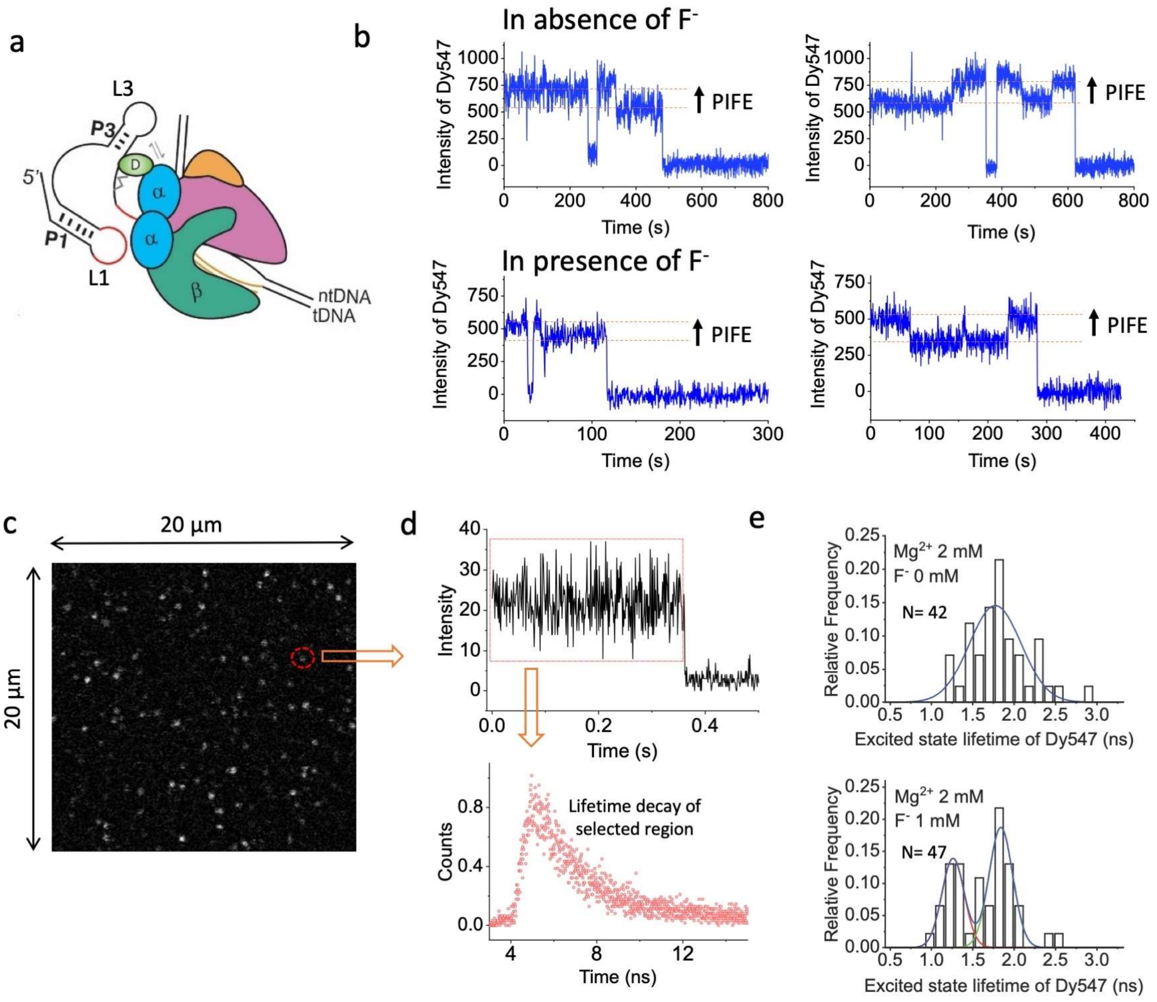

Supplementary Figure 14. Change in micro-environment upon binding of F^- to EC58n (a) A schematic representation of EC58n labeled with a single fluorophore (DY547) at A39. (b) Representative DY547 intensity traces exhibiting PIFE in the absence (top two) and presence (bottom two) of F^-. (c-e) Excited state fluorescence lifetime measurements for single EC58n complexes. (c) Representative laser scanning confocal image of immobilized EC58n complexes. (d) Procedure for extracting the fluorescence lifetime: a time window without any bleaching or blinking events is selected from the photon count time traces (top). This gets converted into a decay profile (bottom), which was best fitted upon deconvolution with the IRF to extract the excited state lifetime of the dye. (e) Lifetime distribution in the presence of 2 mM Mg^{2+} and the absence and presence of F^- as indicated. Two lifetime populations are observed in the presence of F^-.

Supplementary Table 1. Observed kinetic parameters. Errors represent the standard deviation of two independent datasets.

Isolated RNA (RNA64)						
	Low- to mid-FRET		High- to mid-FRET		% SD traces	
	k_{dock} (s^{-1})	k_{undock} (s^{-1})	k_{dock} (s^{-1})	k_{undock} (s^{-1})	0mM F^-	1mM F^-
0 mM Mg^{2+}	1.8 ± 0.2	3.6 ± 0.6	2.6 ± 0.2	3.0 ± 0.2	0	0
1 mM Mg^{2+}	1.8 ± 0.2	0.5 ± 0.0	1.2 ± 0.3	0.6 ± 0.1	5	32
2 mM Mg^{2+}	1.4 ± 0.1	0.5 ± 0.2	0.5 ± 0.2	0.6 ± 0.3	10	52
5 mM Mg^{2+}	1.1 ± 0.0	0.5 ± 0.4	0.9 ± 0.3	0.4 ± 0.1	32	63
10 mM Mg^{2+}	0.7 ± 0.1	0.4 ± 0.3	1.3 ± 0.5	0.5 ± 0.3	40	
2 mM Mg^{2+} + 1mM F^-	2.1 ± 0.1	1.3 ± 0.3	0.6 ± 0.1	0.7 ± 0.5		
RNA in elongation complex (EC64)						
2 mM Mg^{2+}	3.6 ± 0.2	0.5 ± 0.2	1.7 ± 1.2	0.3 ± 0.1	28	69

	$\Delta\Delta G^{0}_{dock}$ (kcal/mol)	$\Delta\Delta G^{++}_{dock}$ (kcal/mol)
RNA64/EC64 (2mM Mg^{2+}) *	0.61	0.58

Equations used to compute energies (see methods);

* $\Delta\Delta G^{0}_{dock} = \Delta G^{0,RNA64}_{dock} - \Delta G^{0,EC64}_{dock}$ and $\Delta\Delta G^{++}_{dock} = -RTln(k^{RNA64}_{dock}/k^{EC64}_{dock})$.

Supplementary Table 2. Oligonucleotides used in this study. The underlined sequences are complementary to allow for surface or transcription bubble capture.

**Dye-labeled RNA oligonucleotides**	
RNA oligonucleotide-1	5’-(Cy5-9S-U)AG GCG AUG GAG UUC GCC AUA AAC GCU GC(5-LC-N-U) UAG CUA AU-3’
RNA oligonucleotide-2 “n”	5’-(N3-U)AG GCG AUG GAG UUC GCC AUA AAC GCU GCU UAG CU(A[2'Dy547]) AU-3’
**Non-labeled RNA oligonucleotides**	
EC RNA48p	5’- pCAC ACG A-3’
EC RNA58	5’- pGAC UCC UAA CCA CAC GA-3’
EC RNA64	5’- pGAC UCC UAC CAG UAA CCA CAC GA-3’
EC RNA64 mutant ‘U48A’	5’- pGAC UCC AAC CAG UAA CCA CAC GA-3’
EC RNA58n mutant ‘M19’	5’-pGAC AUC UAA CCA CAC GA-3’
**Splint DNA/ LNA**	
Splint LNA48p	5’-TC+G +TG+T GAT TAG CTA AGC AGC GTT TAT-3’
Splint DNA58	5’-TCG TGT GGT TAG GAG TCA TTA GCT AAG CAG CGT TTA T-3’
Splint DNA64	5’-GTG GTT ACT GGT AGG AGT CAT TAG CTA AGC AGC GTT TAT-3’
Splint DNA64 mutant ‘U48A’	5’-GTG GTT ACT GGT TGG AGT CAT TAG CTA AGC AGC GTT TAT-3’
Splint DNA58n mutant ‘M19’	5’-TCG TGT GGT TAG ATG TCA TTA GCT AAG CAG CGT TTA T-3’
**Other oligonucleotides**	
tDNA for RNA48p	5’-CTC TGA ATC TCT TCC TCG TGT GAT TCC AAC TTG CTG GGA-3’
tDNA for RNA58, RNA64	5’-CTC TGA ATC TCT TCC TCG TGT GGT TCC AAC TTG CTG GGA-3’
biotin-ntDNA	5’-TCC CAG CAA GTT GCT GAG CTC TTC GGA AGA GAT TCA GAG CCC CCC CCC CCC CCC C/3Bio/ -3’
ntDNA	5’-TCC CAG CAA GTT GCT GAG CTC TTC GGA AGA GAT TCA GAG-3’
CP LNA for RNA48p	/5Biosg/ T+CGT+G+TGA+TT
CP LNA for RNA58, RNA64	/5Biosg/ T+CG+TG+TGG+TT
10nt LNA complementary to A39-U48	5’-A+GG AG+T +CAT T-3’
L1 block LNA	5’-ACT +CCT-3’
**Oligonucleotides used for *in vitro* transcription assays**	
T7A1-PCR	5’-TCC AGA TCC CGA AAA TTT ATC AAA AAG AGT ATT G-3’
crcB-reverse	5’-TCT CAC CTC TTT AAA TAG CTT GCT CAA AAA AAT AG-3’
LambdaPr-CrcB-FWD	5’-GTG CGT GTT GAC TAT TTT ACC TCT GGC GGT GAT AAT GGT TGC ATC CAG AGG GAC ACC CAG AAG A-3’

Supplementary References

1. Watters, K. E., Strobel, E. J., Yu, A. M., Lis, J. T. & Lucks, J. B. Cotranscriptional folding of a riboswitch at nucleotide resolution. *Nat. Struct. Mol. Biol.* **23**, 1124–1131 (2016).
2. Hwang, H. & Myong, S. Protein induced fluorescence enhancement (PIFE) for probing protein-nucleic acid interactions. *Chem. Soc. Rev.* **43**, 1221–9 (2014).
3. Hwang, H., Kim, H. & Myong, S. Protein induced fluorescence enhancement as a single molecule assay with short distance sensitivity. *Proc. Natl. Acad. Sci. U. S. A.* **108**, 7414–8 (2011).
4. Pan, T. & Sosnick, T. RNA Folding During Transcription. *Annu. Rev. Biophys. Biomol. Struct.* **35**, 161–175 (2006).
5. Perdrizet, G. A., Artsimovitch, I., Furman, R., Sosnick, T. R. & Pan, T. Transcriptional pausing coordinates folding of the aptamer domain and the expression platform of a riboswitch. *Proc. Natl. Acad. Sci.* **109**, 3323–3328 (2012).
