# Supplementary material for: An anionic ligand snap-locks a long-range interaction in a magnesium-folded riboswitch
Source: Nat Commun. 2022 Jan 11;13:207. doi: 10.1038/s41467-021-27827-y (PMC8752731; doi:10.1038/s41467-021-27827-y)
Supplement: Supplementary file 2 — Reporting Summary [file 41467_2021_27827_MOESM2_ESM.pdf]

nature portfolio

Corresponding author(s): Nils G. Walter

Last updated by author(s): Nov 12, 2021

Reporting Summary

Nature Portfolio wishes to improve the reproducibility of the work that we publish. This form provides structure for consistency and transparency in reporting. For further information on Nature Portfolio policies, see our Editorial Policies and the Editorial Policy Checklist.

Statistics

For all statistical analyses, confirm that the following items are present in the figure legend, table legend, main text, or Methods section.

n/a	Confirmed	
☐	☒	The exact sample size (*n*) for each experimental group/condition, given as a discrete number and unit of measurement
☐	☒	A statement on whether measurements were taken from distinct samples or whether the same sample was measured repeatedly
☒	☐	The statistical test(s) used AND whether they are one- or two-sided<br>*Only common tests should be described solely by name; describe more complex techniques in the Methods section.*
☒	☐	A description of all covariates tested
☒	☐	A description of any assumptions or corrections, such as tests of normality and adjustment for multiple comparisons
☐	☒	A full description of the statistical parameters including central tendency (e.g. means) or other basic estimates (e.g. regression coefficient) AND variation (e.g. standard deviation) or associated estimates of uncertainty (e.g. confidence intervals)
☒	☐	For null hypothesis testing, the test statistic (e.g. *F*, *t*, *r*) with confidence intervals, effect sizes, degrees of freedom and *P* value noted<br>*Give P values as exact values whenever suitable.*
☒	☐	For Bayesian analysis, information on the choice of priors and Markov chain Monte Carlo settings
☒	☐	For hierarchical and complex designs, identification of the appropriate level for tests and full reporting of outcomes
☒	☐	Estimates of effect sizes (e.g. Cohen's *d*, Pearson's *r*), indicating how they were calculated

Our web collection on statistics for biologists contains articles on many of the points above.

Software and code

Policy information about availability of computer code

Data collection	Labview, Matlab (2015a and 2018b)
Data analysis	Matlab (2015a and 2018b), IDL (Research Systems), QuB, OriginLab (OriginPro 8.5 and 9.1), Image Lab (BIO-RAD)

For manuscripts utilizing custom algorithms or software that are central to the research but not yet described in published literature, software must be made available to editors and reviewers. We strongly encourage code deposition in a community repository (e.g. GitHub). See the Nature Portfolio guidelines for submitting code & software for further information.

Data

Policy information about availability of data

All manuscripts must include a data availability statement. This statement should provide the following information, where applicable:

- Accession codes, unique identifiers, or web links for publicly available datasets
- A description of any restrictions on data availability
- For clinical datasets or third party data, please ensure that the statement adheres to our policy

The data underlying Figs 2c, d, e and 4d, e and Supplementary Fig 1c and Supplementary Fig 4 and Supplementary Fig 6 are provided as a Source Data file and in supplementary table 1. Study data have been deposited in the University of Michigan Deep Blue Data repository (DOI: TBA).

Field-specific reporting

Please select the one below that is the best fit for your research. If you are not sure, read the appropriate sections before making your selection.

☒ Life sciences ☐ Behavioural & social sciences ☐ Ecological, evolutionary & environmental sciences

For a reference copy of the document with all sections, see nature.com/documents/nr-reporting-summary-flat.pdf

Life sciences study design

All studies must disclose on these points even when the disclosure is negative.

Sample size	For smFRET histograms the sample size is the number of molecules in each condition. Different numbers of molecules (ranges in general 50 - 800 molecules) were analyzed for different experimental conditions for getting better estimates of the parameters of interests from a minimum of two movies recorded on different days. For kinetic parameters at least two movies from different days were used to check for reproducibility. For bulk experiments (single-round transcription assay) at least two independent replicates were performed.
Data exclusions	No data were excluded.
Replication	To verify their reproducibility, all of the experiments were repeated at least two times on multiple days, and the replicative experiments were successful.
Randomization	All the experimental conditions were defined before performing the experiment. No specific method for randomization was used.
Blinding	Blinding was not performed as it is not relevant for this study. All recorded movies for smFRET were processed in a similar way using Matlab codes.

Reporting for specific materials, systems and methods

We require information from authors about some types of materials, experimental systems and methods used in many studies. Here, indicate whether each material, system or method listed is relevant to your study. If you are not sure if a list item applies to your research, read the appropriate section before selecting a response.

Materials & experimental systems

n/a	Involved in the study
☒	☐ Antibodies
☒	☐ Eukaryotic cell lines
☒	☐ Palaeontology and archaeology
☒	☐ Animals and other organisms
☒	☐ Human research participants
☒	☐ Clinical data
☒	☐ Dual use research of concern

Methods

n/a	Involved in the study
☒	☐ ChIP-seq
☒	☐ Flow cytometry
☒	☐ MRI-based neuroimaging
